# Potential ER tubular lumen sensing by intrinsically disordered regions

**DOI:** 10.1242/jcs.263696

**Published:** 2025-03-12

**Authors:** Tomohiro Yorimitsu, Ken Sato

**Affiliations:** Department of Life Sciences Graduate School of Arts and Sciences, University of Tokyo, Tokyo 153-8902, Japan

**Keywords:** ER tubules, ER lumen, Sed4, Intrinsically disordered region, ER exit site

## Abstract

Intrinsically disordered regions (IDRs) are known to sense the positive membrane curvature of vesicles and tubules. However, whether IDRs can sense the negative curvature of their luminal surfaces remains elusive. Here, we show that IDRs direct specific localization to endoplasmic reticulum (ER) tubules. In *Saccharomyces cerevisiae*, Sed4 interacts with Sec16 at the ER exit site (ERES) to promote ER export. Upon loss of this interaction, Sed4 failed to assemble at the ERES but was enriched in the ER tubules in a luminal region-dependent manner. Fusion of the Sed4 luminal region with Sec12 and Sec22, which localize throughout the ER, resulted in their enrichment in the tubules. The luminal regions of Sed4 or its homologs, predicted to be IDRs, localized to tubules when translocated alone into the ER lumen. The lumen-imported IDRs derived from cytosol-localizing Sec16 and Atg13 also exhibited tubule localization. Furthermore, Sed4 constructs in which the luminal region was replaced by these IDRs were concentrated at the ERES. Collectively, we suggest that the IDRs sense the properties of the tubule lumen, such as its surface, and facilitate Sed4 assembly at the ERES.

## Teaser

**Highlighted Article:** Intrinsically disordered regions of the Sed4 ER membrane protein family and the cytosolic proteins Sec16 and Atg13 localize to tubules when imported into the ER lumen, suggesting their ability to sense specific features of the tubular lumen.

## INTRODUCTION

The endoplasmic reticulum (ER) is a membranous organelle composed of a continuous network of tubular and sheet-like structures (Obara et al., 2023; Shibata et al., 2009). This architecture is formed by evolutionarily conserved ER-shaping proteins. The highly curved tubules of the ER are generated and stabilized by transmembrane proteins known as reticulons and DP1 family proteins (De Craene et al., 2006; Voeltz et al., 2006). In *Saccharomyces cerevisiae*, Rtn1, Rtn2 and Yop1 specifically localize to the ER tubules and are involved in ER tubule generation and stabilization. Disruption of these proteins collapses the ER network, causing a significant reduction in tubules and expansion of sheets (De Craene et al., 2006; Voeltz et al., 2006).

Protein biosynthesis is one of the main functions of the ER. Some newly synthesized proteins in the ER exit to enter the secretory pathway, which utilizes COPII transport vesicles or carriers generated by the COPII protein at the ER subdomains termed ER exit sites (ERESs) (Kurokawa and Nakano, 2019; Raote et al., 2023). The ERES is a conserved transport system among yeast, protozoa, insects, mammals and plants. In these cells, the ERES has been observed by fluorescence microscopy as the site where the fluorescent protein-tagged COPII protein assembles (daSilva et al., 2004; Hammond and Glick, 2000; Ivan et al., 2008; Rossanese et al., 1999; Sealey-Cardona et al., 2014). Although the mechanisms underlying ERES formation are not fully understood, ERESs are found in highly curved membrane domains, such as tubules and sheet edges (Okamoto et al., 2012; Stadler et al., 2018). Sec16 has long been suggested as a key player in ERES generation (Bhattacharyya and Glick, 2007; Connerly et al., 2005; Espenshade et al., 1995; Hughes et al., 2009). In the COPII vesicle formation reaction, the ER membrane protein Sec12 acts as a GTP-GDP exchange factor for the small GTPase Sar1, allowing GTP-bound Sar1 to bind to the ER membrane. The COPII coat complex is then recruited to the membranes by binding to GTP-bound Sar1. These COPII components interact with Sec16 to assemble at the ERES (Barlowe et al., 1993; Matsuoka et al., 1998; Nakano and Muramatsu, 1989; Shaywitz et al., 1997; Supek et al., 2002; Yorimitsu and Sato, 2012). Recently, mammalian Sec16 was shown to assemble as a condensate through its N-terminal intrinsically disordered region (IDR), which drives the formation of a liquid-like ERES by liquid–liquid phase separation (LLPS). The liquid-like state of ERES, regulated by dual-specificity tyrosine-regulated kinase 3 (DYRK3) and protein phosphatase 1 (PP1), is essential for proper ER export (Gallo et al., 2023).

In *S. cerevisiae*, the Sec12 homolog Sed4 is involved in COPII vesicle formation (Gimeno et al., 1995). The cytosolic domains of Sec12 and Sed4 share 45% amino acid identity. The cytosolic domain of Sec12 has been crystallographically shown to fold into a β-propeller structure (Joiner and Fromme, 2021; McMahon et al., 2012), and the cytosolic domain of Sed4 is predicted to have a structure similar to that of Sec12 (Schlacht and Dacks, 2015). Despite this similarity, Sed4 has no GTP-GDP exchange activity for Sar1 (Kodera et al., 2011; Saito-Nakano and Nakano, 2000). Furthermore, Sed4 interacts with Sec16 through its cytosolic domain, whereas Sec12 does not (Gimeno et al., 1995). We recently discovered that this differential ability to interact with Sec16 leads to different distribution patterns of Sec12 and Sed4 (Yorimitsu and Sato, 2023). Sec12 localizes throughout the ER with no concentration at the ERES, whereas Sed4 is enriched at the ERES together with Sec16.

The luminal region of Sed4 is much longer, consisting of 696 amino acids, compared to the 97 amino acids of Sec12. In addition to its cytosolic domain, Sed4 requires its luminal region for assembly at the ERES (Yorimitsu and Sato, 2023). The luminal region of Sed4 has been shown to undergo multiple *O*-mannosyl modifications (Neubert et al., 2016). We found that Sed4 oligomerizes through its luminal region and that *O*-mannosylation is involved in stabilizing the oligomer (Yorimitsu and Sato, 2023). The long luminal region is a characteristic feature of Sed4 orthologs in *Saccharomyces* species and *Candida glabrata* (Schlacht and Dacks, 2015). In contrast to *S. cerevisiae* Sec12, *Pichia pastoris* Sec12 (PpSec12) possesses a long luminal domain, which is involved in its oligomerization and localization to the ERES (Soderholm et al., 2004). However, there is no homology in the luminal regions between Sed4 and PpSec12, or among the Sed4 orthologs, although all these proteins have an ER retention signal at the end of their luminal regions. The mechanism underlying the assembly of Sed4 and PpSec12 at the ERES via the luminal region remains to be elucidated.

Our recent study showed that, as interaction with Sec16 is essential for Sed4 to assemble at the ERES, loss of this interaction prevents Sed4 from assembling at the ERES (Yorimitsu and Sato, 2023). However, under these conditions, Sed4 did not uniformly localize to the ER, but was significantly enriched in the ER tubules. These results suggest that Sed4 has a tubule-targeting mechanism. Here, we show that the luminal region of Sed4 senses the ER tubular lumen. The luminal regions of Sed4 and its homologs PpSec12 and *C. glabrata* Sed4 (CgSed4) were found to exclusively localize to tubules when translocated into the ER lumen, indicating that this action is independent of the transmembrane domain. These luminal regions are predicted to be intrinsically disordered. Notably, we discovered that Sed4-unrelated IDRs derived from the cytosolic proteins Sec16 and autophagy-related protein 13 (Atg13) can sense the tubular lumen. Finally, we show that the IDR of the Sed4 luminal region drives its assembly at the ERES, suggesting that intrinsic disorder plays a role in the physiological function of Sed4.

## RESULTS

### The luminal region of Sed4 functions in targeting to ER tubules

**Fig. 1.**
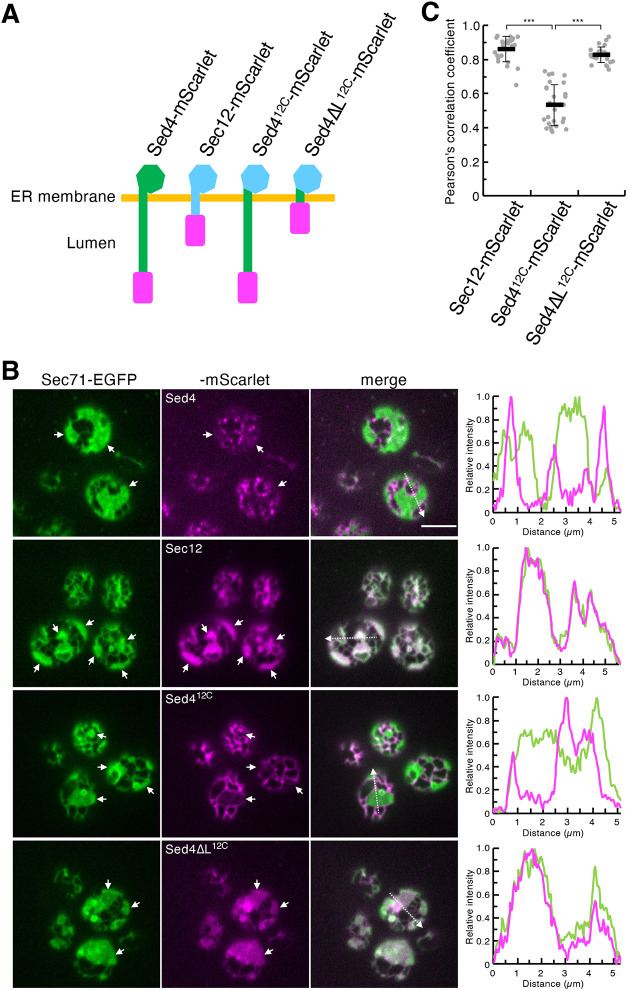
**The luminal region of Sed4 is required for the localization of Sed4^12C^ to ER tubules.** (A) Schematic diagram of the fusion constructs used for fluorescence microscopy. The constructs have mScarlet (shown in magenta) fused to their C-terminal ends. In the chimeric constructs, the Sed4- and Sec12-derived portions are shown in green and light blue, respectively. (B) *rtn1*Δ *rtn2*Δ *yop1*Δ cells expressing Sec71–EGFP with Sed4–mScarlet, Sec12–mScarlet, Sed4^12C^–mScarlet or Sed4ΔL^12C^–mScarlet were grown to mid-log phase. Images were obtained using a confocal fluorescence microscope by focusing on the periphery of the cells. White arrows represent ER sheets. The intensity profile plots along the white dashed arrows in the merged images are shown in the right panels. Scale bar: 4 μm. (C) Quantification of Pearson's correlation coefficients for individual mScarlet constructs and Sec71–EGFP (at least 25 cells from three independent experiments). Error bars represent standard deviation. *P*-values were calculated by unpaired two-tailed *t*-test. ***P<0.001.

To observe the ER network in *S. cerevisiae* by fluorescence microscopy, *rtn1*Δ *rtn2*Δ *yop1*Δ cells were used as described previously (Voeltz et al., 2006). In these cells, the network of sheet and tubular structures is more clearly observed when labeled with a fluorescent protein-tagged ER marker protein. This is because the sheets are expanded compared to those in wild-type cells, in which they are rarely visible. When the ER marker Sec71–EGFP was visualized, the ER network was observed by focusing on the periphery of the cells as shown in Fig. 1. To examine the role of the luminal region of Sed4 in its assembly at the ERES, we co-expressed the C-terminally mScarlet-tagged constructs with Sec71–EGFP (Fig. 1A). Sed4–mScarlet formed dot-like structures localized to highly curved membrane regions of the ER, such as tubular regions and edges of the sheets, as Sed4–mScarlet was previously reported to be concentrated at the ERES (Yorimitsu and Sato, 2023). In contrast, Sec12–mScarlet did not show any concentration at the ERES and its distribution pattern overlapped with that of Sec71–EGFP (Fig. 1B). For assembly at the ERES, Sed4 was shown to require interaction with Sec16 through its cytosolic domain (Yorimitsu and Sato, 2023). Although it shares an amino acid sequence with Sed4 in its cytosolic domain, Sec12 does not interact with Sec16 (Gimeno et al., 1995; Yorimitsu and Sato, 2023). In line with these observations, Sed4^12C^–mScarlet, in which the cytosolic domain of Sed4 was replaced by the corresponding domain of Sec12 (Fig. 1A), was not concentrated at the ERES. However, in contrast to Sec12–mScarlet, Sed4^12C^–mScarlet was enriched in ER tubules, as shown previously (Fig. 1B and C, Yorimitsu and Sato, 2023). To confirm the role of the luminal region of Sed4 in this distribution, we truncated the luminal region of Sed4^12C^ and generated Sed4ΔL^12C^–mScarlet (Fig. 1A). Similar to Sec12, Sed4ΔL^12C^–mScarlet was not enriched in tubules and, instead, largely colocalized with Sec71–EGFP throughout the ER (Fig. 1B,C). These results demonstrate that the luminal region of Sed4 is required for the predominant localization of Sed4^12C^ to tubules.

**Fig. 2.**
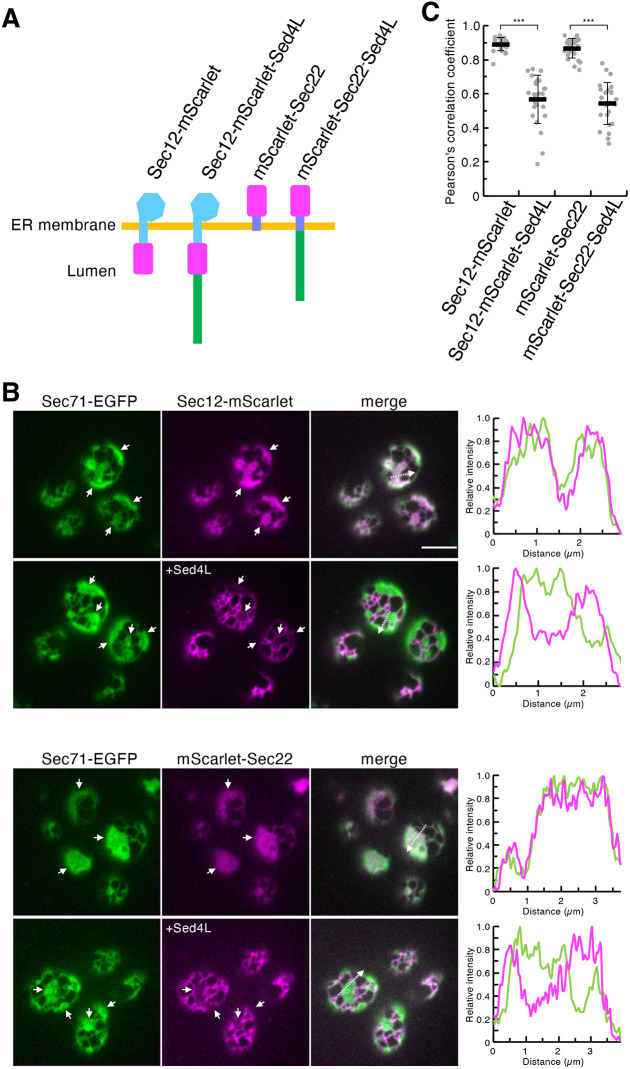
**Fusion of the luminal region of Sed4 to Sec12 and Sec22 enriches them in ER tubules.** (A) Schematic diagram of the fusion constructs used for fluorescence microscopy. mScarlet (shown in magenta) was fused to the C-terminal end of Sec12 and to the N-terminal end of Sec22 to generate Sec12–mScarlet and mScarlet–Sec22, respectively. In the Sec12–mScarlet–Sed4L and mScarlet–Sec22–Sed4L constructs, the luminal region of Sed4 (shown in green) was fused C-terminally to Sec12–mScarlet and mScarlet–Sec22, respectively. (B) *rtn1*Δ *rtn2*Δ *yop1*Δ cells expressing Sec71–EGFP with Sec12–mScarlet, mScarlet–Sec22, Sec12–mScarlet–Sed4L or mScarlet–Sec22–Sed4L were grown to mid-log phase. The Sed4L-fused constructs are indicated by ‘+Sed4L’. Images were obtained using a confocal fluorescence microscope by focusing on the periphery of the cells. White arrows represent ER sheets. The intensity profile plots along the white dashed arrows in the merged images are shown in the right panels. Scale bar: 4 μm. (C) Quantification of Pearson's correlation coefficients for individual mScarlet constructs and Sec71–EGFP (at least 25 cells from three independent experiments). Error bars represent standard deviation. *P*-values were calculated by unpaired two-tailed *t*-test. ***P<0.001.

We then examined whether the luminal region of Sed4 (Sed4L) mediated tubular ER localization. To this end, in addition to Sec12–mScarlet, mScarlet–Sec22 was first generated by tagging the N-terminus of Sec22 with mScarlet (Fig. 2A). Similar to Sec12–mScarlet, mScarlet–Sec22 colocalized with Sec71–EGFP throughout the ER, as observed previously (Date et al., 2022) (Fig. 2B). The Sed4L sequence was then added to the C-termini of these fusion constructs to yield Sec12–mScarlet–Sed4L and mScarlet–Sec22–Sed4L (Fig. 2A). In contrast to the constructs without Sed4L fusion, Sec12–mScarlet–Sed4L and mScarlet–Sec22–Sed4L were found to be enriched in the tubules (Fig. 2B,C). These results suggest that Sed4L plays a role in tubule targeting.

### ER lumen-imported Sed4L resides in tubules

**Fig. 3.**
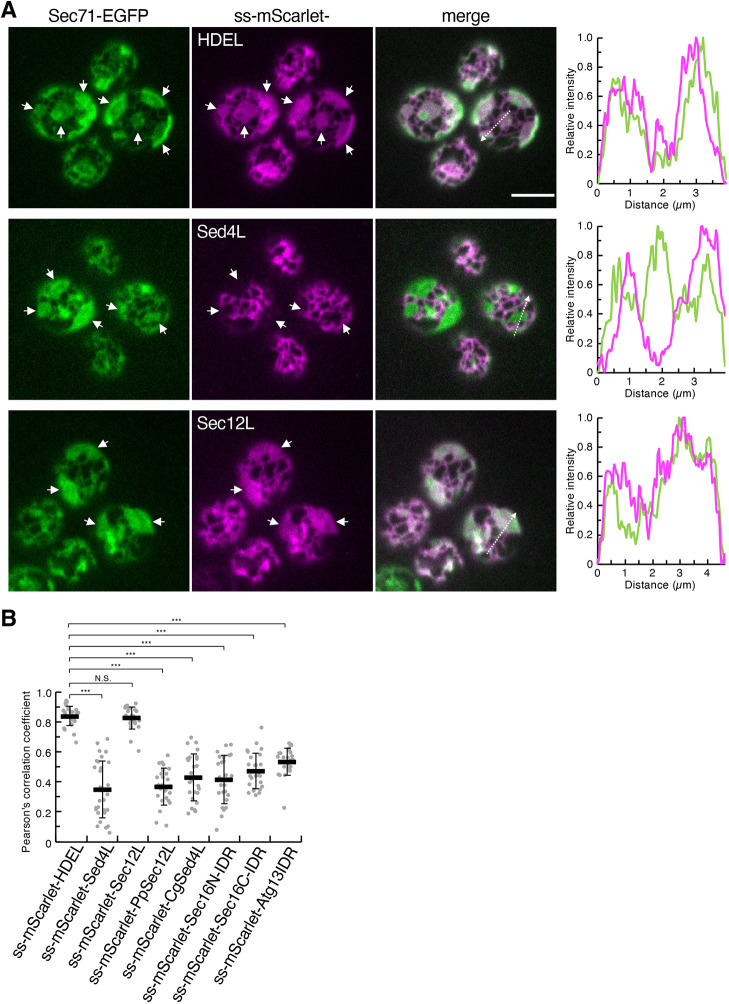
**The luminal region of Sed4 specifically localizes to tubules when translocated into the ER lumen.** (A) *rtn1*Δ *rtn2*Δ *yop1*Δ cells expressing Sec71–EGFP with ss–mScarlet–HDEL, ss–mScarlet–Sed4L or ss–mScarlet–Sec12L were grown to mid-log phase. Images were obtained using a confocal fluorescence microscope by focusing on the periphery of the cells. White arrows represent ER sheets. The intensity profile plots along the white dashed arrows in the merged images are shown in the right panels. Scale bar: 4 μm. (B) Quantification of Pearson's correlation coefficients for individual ss–mScarlet constructs and Sec71–EGFP (at least 25 cells from three independent experiments). Error bars represent standard deviation. *P*-values were calculated by unpaired two-tailed *t*-test. N.S., not significant, *P*>0.05; ***P<0.001.

Next, we investigated whether Sed4L could be targeted to the tubules independently of the transmembrane domain. To address this question, we generated ss–mScarlet–HDEL and ss–mScarlet–Sed4L. In these constructs, the Kar2 signal sequence (ss) was added to the N-terminus of mScarlet (ss–mScarlet) and then its C-terminus was fused to the ER retention signal HDEL and Sed4L, respectively. As a result, these constructs could be translocated into the ER lumen and behave like luminal proteins. Furthermore, ss–mScarlet–Sec12L was produced by fusing ss–mScarlet to the luminal region of Sec12 (Sec12L). The HDEL sequence, which Sed4 endogenously possesses, was added to the C-terminus of ss–mScarlet–Sec12L. These constructs were observed with Sec71–EGFP in *rtn1*Δ *rtn2*Δ *yop1*Δ cells (Fig. 3A). Expectedly, ss–mScarlet–HDEL colocalized with Sec71–EGFP throughout the ER. ss–mScarlet–Sec12L also fully stained both ER tubules and sheets. In contrast to these constructs, ss–mScarlet–Sed4L showed a distribution pattern distinct from that of Sec71–EGFP, being enriched in the tubules (Fig. 3B). These results indicate that Sed4L can localize to the tubules independently of the transmembrane domain. No differences in the morphology and location of ERES were observed between cells expressing the ERES marker Sec16–tdTomato with ss–mScarlet–HDEL and ss–mScarlet–Sed4L, indicating that the tubule localization of ss–mScarlet–Sed4L does not affect ERES formation (Fig. S1).

To determine the functional part of Sed4L required for tubule localization, a series of truncation mutants were generated and visualized as described above. Among the ss–mScarlet–Sed4L mutants, Δ470–1061, Δ570–1061, Δ370–769, Δ370–869 and Δ370–969 showed localization throughout the ER (Fig. S2A). Because the amino acid sequence 570–769 is a common region missing in these constructs, it was suggested that this region plays a role in tubule localization. However, further analysis revealed that all ss–mScarlet–Sed4L truncation mutants related to the 570–769 amino acid region, such as Δ670–869, Δ570–769, Δ469–679, Δ669–970, Δ570–869 and Δ470-779, localized to tubules in a manner comparable to full-length ss–mScarlet–Sed4L. These results suggest that tubule localization requires the entire Sed4L or multiple parts within it, rather than a single segment.

Sed4 is highly *O*-mannosylated in the luminal region. 55 *O*-mannosylated serine and threonine residues have been identified using glycoproteomic analysis (Neubert et al., 2016). In our previous study, the Sed4ΔOM mutant was created by replacing these residues with alanine and its oligomerization was shown to be partially destabilized (Yorimitsu and Sato, 2023). However, the *O*-mannosylation-defective construct ss–mScarlet–Sed4LΔOM was found to localize to tubules in a manner comparable to ss–mScarlet–Sed4L (Fig. S2B), indicating that *O*-mannosylation is dispensable for tubule localization.

### Luminal regions of Sed4 and its homologs PpSec12 and CgSed4 are intrinsically disordered and can sense the ER tubular lumen

**Fig. 4.**
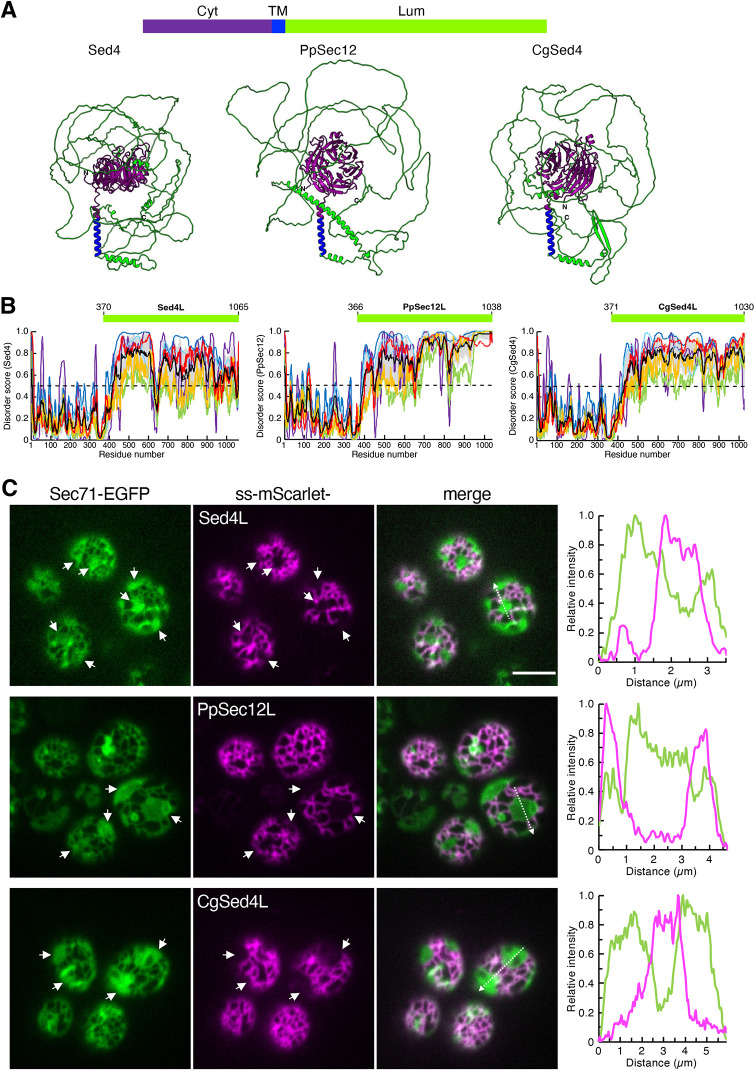
**The intrinsically disordered luminal regions of Sed4 and its homologs PpSec12 and CgSed4 localize to tubules when translocated into the ER lumen.** (A) AlphaFold-predicted structures of Sed4, PpSec12 and CgSed4. In the schematic diagram, the cytoplasmic (Cyt), transmembrane (TM) and luminal (Lum) regions are represented in purple, blue and green, respectively. The colored regions correspond to those in the predicted structures. (B) Disorder profiles of Sed4, PpSec12 and CgSed4 sequences were generated using the disorder predictor Rapid Intrinsic Disorder Analysis Online (RIDAO; https://ridao.app). Each line represents the value of the prediction program: ‘PONDR VLXT’ (purple), ‘PONDR VLS2’ (blue), ‘PONDR VL3’ (cyan), ‘PONDR VLS2’ (red), ‘IUPred-Short’ (green), ‘IUPred-Long’ (orange) or ‘PONDR FIT’ (red). The mean disorder profile (black line) was generated by averaging the individual profiles, with corresponding errors shown in light gray shading. The green rectangles above the disorder profiles represent the protein regions with the amino acid numbers used in this study. (C) *rtn1*Δ *rtn2*Δ *yop1*Δ cells expressing Sec71–EGFP with ss–mScarlet–Sed4L, ss–mScarlet–PpSec12L or ss–mScarlet–CgSed4L were grown to mid-log phase. Images were observed under a confocal fluorescence microscope by focusing on the periphery of the cell. White arrows represent the ER sheets. The intensity profile plots along a white dashed arrow in the merged images are shown in the right panels. Images represent three independent experiments. Scale bar: 4 μm.

Although its structure has not been experimentally determined, the predicted structure of Sed4 is available in the AlphaFold database (Jumper et al., 2021). The cytosolic domain is folded into a β-propeller structure, as proposed previously (Schlacht and Dacks, 2015), whereas the luminal region is predicted to have no strong structural signature and is mostly unstructured (Fig. 4A). Furthermore, AlphaFold provided the structures of the two Sed4 homologs, PpSec12 and CgSed4 (Fig. 4A). In the amino acid sequences of the cytosolic domain, *S. cerevisiae* Sed4 exhibits 35% and 49% identity to PpSec12 and CgSed4, respectively. This relatively high degree of homology is consistent with the AlphaFold structural prediction that the cytosolic domains of PpSec12 and CgSed4 are folded into a structure similar to that of Sed4. However, the luminal regions of these two proteins are poorly homologous to that of Sed4, sharing only less than 20% sequence identity. Nevertheless, similar to Sed4L, the luminal regions of PpSec12 and CgSed4 are predicted to be largely unstructured. We further analyzed the amino acid sequences of the luminal regions of Sed4, PpSec12 and CgSed4 using the web-based IDR predictor Rapid Intrinsic Disorder Analysis Online (RIDAO) (Dayhoff and Uversky, 2022). Bioinformatic analysis revealed a highly disordered score for all luminal regions compared to the score for the cytosolic domains (Fig. 4B). Based on these observations, we concluded that Sed4, PpSec12 and CgSed4 all have intrinsically disordered luminal regions.

To test whether, similar to Sed4L, the luminal regions of PpSec12 (PpSec12L) and CgSed4 (CgSed4L) localized to the ER tubular lumen, ss–mScarlet–PpSec12L and ss–mScarlet–CgSed4L were generated by fusing PpSec12L and CgSed4L to the C-terminus of ss–mScarlet, as described above. These constructs were visualized with Sec71–EGFP in *rtn1*Δ *rtn2*Δ *yop1*Δ cells by fluorescence microscopy (Fig. 4C). ss–mScarlet–PpSec12L and ss–mScarlet–CgSed4L were distributed differently from Sec71–EGFP and showed preferential localization to the ER tubules, as observed for ss–mScarlet–Sed4L (Fig. 3B). These results suggest that, in addition to Sed4L, PpSec12L and CgSed4L can specifically recognize the ER tubular lumen. In contrast to ss–mScarlet–Sed4L and ss–mScarlet–PpSec12L, ss–mScarlet–CgSed4L was partially transported to the vacuoles (Fig. S3). At the end of the luminal region, PpSec12 as well as Sed4 have the HDEL sequence, whereas CgSed4 possesses an NDEL sequence. We speculate that the mistrafficking of CgSed4 to the vacuoles occurs because the NDEL sequence of CgSed4 is ineffective as an ER retention signal in *S. cerevisiae*.

### ER lumen-imported IDRs of Sec16 and Atg13 localize to tubules

**Fig. 5.**
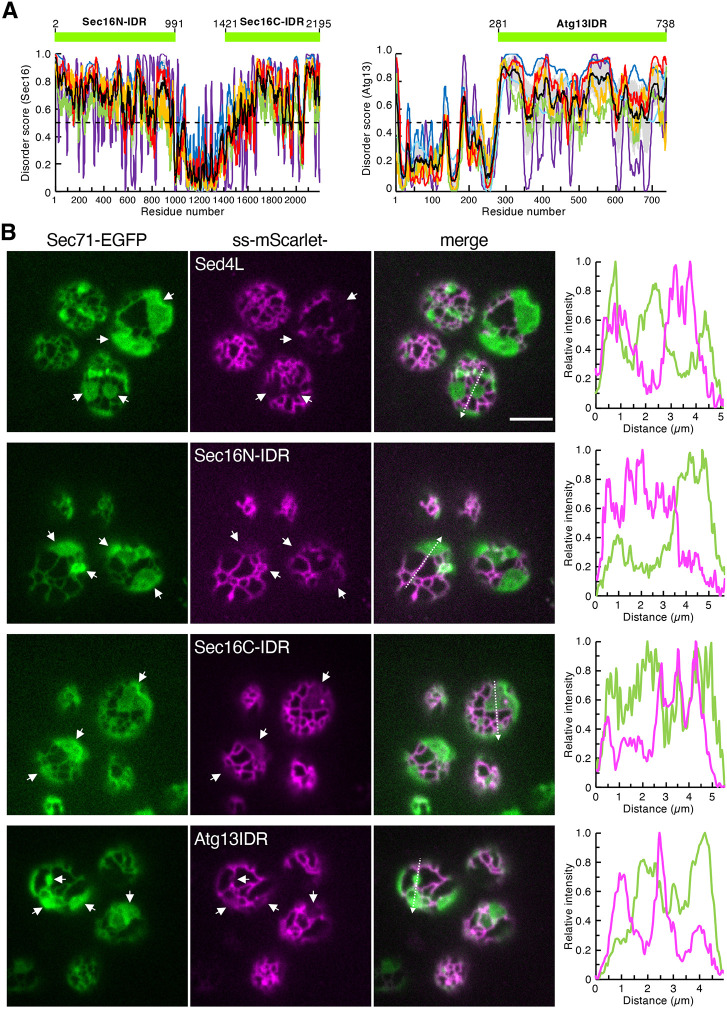
**IDRs derived from the cytosolic proteins Sec16 and Atg13 localize to tubules when translocated into the ER lumen.** (A) Disorder profiles of Sec16 and Atg13 sequences were generated as described in Fig. 4B. The mean disorder profile (black line) was generated by averaging the individual profiles, with corresponding errors shown in light gray shading. The green rectangles above the disorder profiles represent the protein regions with the amino acid numbers used in this study. (B) *rtn1*Δ *rtn2*Δ *yop1*Δ cells expressing Sec71–EGFP with ss–mScarlet–Sed4L, ss–mScarlet–Sec16N-IDR, ss–mScarlet–Sec16C-IDR or ss–mScarlet–Atg13IDR were grown to mid-log phase. Images were observed under a confocal fluorescence microscope by focusing on the periphery of the cell. White arrows represent the ER sheets. The intensity profile plots along a white dashed arrow in the merged images are shown in the right panels. Images represent three independent experiments. Scale bar: 4 μm.

Owing to the low conservation of amino acid sequences among Sed4L, PpSec12L and CgSed4L, we hypothesized that their ability to reside in the tubules is driven by a characteristic intrinsic disorder. To verify this hypothesis, we examined the IDRs of the cytosolic proteins Sec16 and Atg13. Sec16 has two long IDRs at the N- and C-termini (N-IDR and C-IDR, respectively), whereas Atg13 has an IDR at the C-terminal end (Fig. 5A) (Jao et al., 2013; Pietrosemoli et al., 2013). Comparison of the amino acid sequences of Sec16 N-IDR, Sec16 C-IDR and the Atg13 IDR with those of Sed4L revealed only 17% or less identity. As described above, ss–mScarlet was fused C-terminally to the Sec16 N-IDR and C-IDR to generate ss–mScarlet–Sec16N-IDR and ss–mScarlet–Sec16C-IDR, respectively. ss–mScarlet–Atg13IDR was constructed by fusing the Atg13 IDR with the C-terminus of ss–mScarlet. These three constructs contained an HDEL sequence added at each C-terminus. When expressed with Sec71–EGFP in *rtn1*Δ *rtn2*Δ *yop1*Δ cells, they were observed to label the ER. However, all constructs showed a different distribution pattern from that of Sec71–EGFP, which exclusively localized to the tubules (Figs 3B and 5B), similar to ss–mScarlet–Sed4L. These results suggest that even the IDRs derived from cytosolic proteins unrelated to Sed4 can sense the tubular ER lumen.

### IDR drives Sed4 assembly at the ERES

**Fig. 6.**
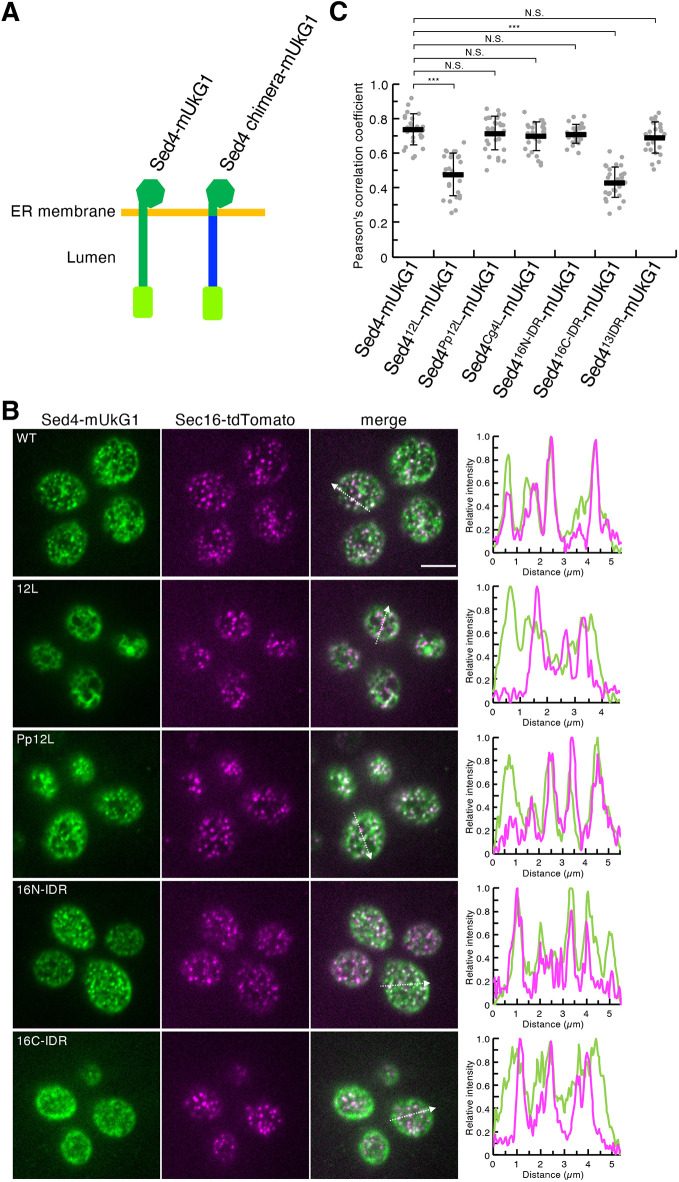
**IDRs can function in a manner similar to Sed4L in the assembly of Sed4 at the ERES.** (A) Schematic diagram of the mUkG1 fusion constructs used for fluorescence microscopy. The mUkG1 moiety is shown in light green. In Sed4 chimera–mUkG1, the luminal region of Sed4 was replaced by the protein region (shown in blue) corresponding to Sec12L, PpSec12L, CgSed4L, Sec16N-IDR, Sec16C-IDR or Atg13IDR. (B) Cells expressing Sec16–tdTomato with Sed4–mUkG1, Sed4^12L^–mUkG1, Sed4^Pp12L^–mUkG1, Sed4^16N-IDR^–mUkG1 or Sed4^16C-IDR^–mUkG1 were grown a mid-log phase. Images were obtained using a confocal fluorescence microscope by focusing on the periphery of the cells. The intensity profile plots along the white dashed arrows in the merged images are shown in the right panels. Scale bar: 4 μm. (B) Quantification of Pearson's correlation coefficients for individual mUkG1 constructs and Sec16–tdTomato (at least 26 cells from three independent experiments). Error bars represent standard deviation. *P*-values were calculated by unpaired two-tailed *t*-test. N.S., not significant, *P*>0.05; ***P<0.001.

Sed4 tagged with the fluorescent protein mUkG1 (Sed4–mUkG1, Fig. 6A) was concentrated at the ERES with Sec16–tdTomato (Fig. 6B), as shown previously (Yorimitsu and Sato, 2023). In this study, we found that the assembly of Sed4 at the ERES requires the luminal region. In line with this observation, Sed4^12L^–mUkG1, in which Sed4L was replaced with Sec12L (Fig. 6A), localized throughout the ER without assembling at the ERES (Fig. 6B,C). We then investigated whether PpSec12L and CgSed4L act similar to Sed4L to mediate the assembly of Sed4 at the ERES. To address this issue, the luminal region of Sed4–mUkG1 was substituted with PpSec12L and CgSed4L to generate Sed4^Pp12L^–mUkG1 and Sed4^Cg4L^–mUkG1, respectively (Fig. 6A). When visualized with Sec16–tdTomato, Sed4^Pp12L^–mUkG1 and Sed4^Cg4L^–mUkG1 showed dot-like structures that colocalized with Sec16–tdTomato (Fig. 6B,C; Fig. S4), indicating that the Sed4^Pp12L^ and Sed4^Cg4L^ constructs assembled at the ERES because PpSec12L and CgSed4L act similar to Sed4L. These results suggest that the IDR property is crucial for the role of the luminal region in Sed4 assembly at the ERES. To test this possibility, we replaced Sed4L with Sec16 N-IDR, Sec16 C-IDR and the Atg13 IDR to generate Sed4^16N-IDR^–mUkG1, Sed4^16C-IDR^–mUkG1 and Sed4^13IDR^–mUkG1, respectively. Similar to Sed4–mUkG1, Sed4^16N-IDR^–mUkG1 and Sed4^13IDR^–mUkG1 were enriched at the ERES together with Sec16–tdTomato (Fig. 6B,C; Fig. S4), although Sed4^13C-IDR^–mUkG1 showed only weak signals and was partially missorted to the vacuolar membrane by an unknown mechanism (Fig. S5). These findings suggest that intrinsic disorder in the luminal region of Sed4 facilitates its assembly at the ERES. Unlike these two constructs, Sed4^16C-IDR^–mUkG1 was not concentrated at the ERES, suggesting that not all IDRs can function as Sed4L.

## DISCUSSION

We previously investigated the interaction between Sed4 and Sec16 and showed that they mutually assemble at the ERES (Yorimitsu and Sato, 2023). In this study, upon loss of the interaction with Sec16, Sed4 failed to be concentrated at the ERES but was significantly enriched in the ER tubules. Sed4 and its homologs have a characteristic long luminal region following a single transmembrane domain. We propose that the luminal region is involved in tubule localization. By analyzing the luminal region-truncated Sed4ΔL^12C^ and the Sed4L-fused Sec12 and Sec22 constructs, we demonstrate here that Sed4L is capable of targeting to tubules.

The ER tubule is a highly curved membrane structure compared with the sheet (Shibata et al., 2009). ER-shaping proteins, reticulons and Yop1 (homologous to receptor expression-enhancing proteins or REEPs), are well characterized for specific localization to the ER tubules, which is mediated by the reticulon homology domain consisting of wedge-like transmembrane domains and a C-terminal amphipathic helix (Brady et al., 2015; Shibata et al., 2008; Voeltz et al., 2006), whereas the tubule-localized soluble ER luminal proteins remain undetermined. In a previous study, the transmembrane domains of Sec12 and Sed4 were shown to play a role in ER localization (Sato et al., 1996). Thus, the transmembrane domain might be involved in the tubule-targeting function of Sed4L. However, this possibility was ruled out by our observation that Sed4L, PpSed4L and CgSed4L exclusively localized to the tubules when translocated into the ER lumen, demonstrating that the tubule localization of these luminal regions is independent of the transmembrane domain.

AlphaFold structural prediction revealed that Sed4L, PpSed4L and CgSed4L are primarily unstructured flexible loops. Consistent with this prediction, bioinformatics analysis of the amino acid sequences indicated that they are intrinsically disordered. These findings challenged our assumption that that these luminal regions might contain structured domains that function in the preferential localization to tubules and suggested that the intrinsic disorder in the luminal region is responsible for this function. This notion is supported by the intriguing finding that tubule localization is not exclusive to the luminal IDRs of the Sed4 family. Despite the cytosolic protein components, Sec16 and Atg13 IDRs also exhibited this behavior in the ER lumen. These observations led us to conclude that intrinsic disorder is a physicochemical property of the protein fragments that perform the tubular lumen sensing. Intrinsically disordered proteins or regions have been shown to coordinate multiple sites for their functions (Fujioka et al., 2020; Stancheva et al., 2020; Zeno et al., 2019). We have previously shown that Sed4 oligomerization is mediated by the multiple sites in the luminal region (Yorimitsu and Sato, 2023). Therefore, our results for the Sed4L truncation mutants shown here, which indicate that multiple sites of Sed4L are involved in tubule localization, might also reflect the IDR properties of Sed4L.

How do these IDRs specifically localize to the tubule? We propose that the IDRs are capable of sensing specific properties of the tubule luminal environment. One possible mechanism is that the IDRs could sense the lipids that are specific to the tubule bilayers. As lipids are symmetrically distributed between the inner and outer leaflets of the ER membrane bilayer (Pabst and Keller, 2024; Sprong et al., 2001; van Meer et al., 2008), we hypothesized that the cytosolically expressed IDRs localize to the tubules. To test this hypothesis, we visualized a signal sequence-deficient, mNeonGreen-fused Sed4L construct (Fig. S6). This construct showed no specific organelle localization but diffused in the cytosol, suggesting that Sed4L does not specifically sense tubule lipids. Recent quantitative lipidomics analysis revealed that the lipid compositions of ER membranes isolated via the ER tubule-resident protein Rtn1–bait and via the sheet-abundant perinuclear ER membrane protein Elo3–bait are nearly identical (Reinhard et al., 2024). This finding suggests that, despite their structural differences, tubular and sheet domains share similar lipid compositions. These observations suggest that the lipid specificity of the tubules does not serve as a trigger for Sed4L localization, although current studies on the lipid composition of ER domains remain limited.

Another possibility is that the IDRs are recruited to the tubules by binding to tubule-localizing proteins that have a high affinity for the IDRs in the luminal domain; candidate proteins have not yet been identified. In *S. cerevisiae*, Rtn1, Rtn2 and Yop1 are known to be the major tubule-localizing proteins (De Craene et al., 2006; Voeltz et al., 2006). In this study, the tubule localization of the IDRs was observed in *rtn1*Δ *rtn2*Δ *yop1*Δ, indicating that such localization is not mediated by binding to these proteins. Currently, 18 proteins that interact with Sed4 are listed in the *Saccharomyces* Genome Database (https://www.yeastgenome.org). In addition to these proteins, the ER luminal chaperones have been found to bind to the luminal region of Sed4 (Fan et al., 2024). However, none of these proteins are the tubule-localizing proteins. Taken together, it remains to be determined whether ER proteins, in addition to lipids, contribute to the tubule localization of the IDRs.

Third, it is possible that the tubule lumen-translocated IDRs can recognize the surface of the tubular lumen by potentially sensing its negative (concave) membrane curvature. Curvature-sensing mechanisms have been extensively studied for structured protein domains, such as the reticulon homology domain (Cail and Drubin, 2023; Kozlov and Taraska, 2023). The Bin/amphiphysin/Rvs (BAR) domain is also a membrane curvature sensor that folds into a crescent-like shape and binds to membrane curvatures; F-BAR binds to positive (convex) curvature, whereas I-BAR binds to negative curvature (Mim and Unger, 2012). However, there is no homology between the luminal regions and any known domain, including I-BAR.

IDRs derived from specific endocytic proteins such as epsin 1, AP180 and amphiphysin 1 have been shown to be involved in sensing positive membrane curvature (Busch et al., 2015; Yuan et al., 2023; Zeno et al., 2018). These IDRs require the membrane-binding domain for curvature sensing and simultaneously act as a membrane curvature inducers. In this model, positive membrane curvature has been proposed to be sensed or induced by IDRs through a combination of molecular crowding and entropic and electrostatic mechanisms (Zeno et al., 2019). In contrast, whether IDRs function as negative curvature sensors has not yet been investigated (Johnson et al., 2024). This study suggests the novel function of the IDRs in potentially sensing negative curvature.

In contrast to the observations with the positive curvature-sensing IDRs, we clearly observed that the ER network in cells with the IDR constructs, which do not require the membrane-binding domain for tubule sensing as described above, was morphologically comparable to that in cells with the control ss–mScarlet–HDEL. These results indicate that these constructs cannot promote tubule formation, implying that the IDRs used here do not induce membrane curvature. Therefore, whether these IDRs can really sense negative curvature and whether they use the same mechanisms as those involved in positive curvature sensing remain open questions.

The Sec16 N-IDR and Atg13 IDR have been shown to undergo LLPS. Under the control of DYRK3 and PP1, the Sec16 N-IDR drives LLPS to form liquid-like condensates. This LLPS-mediated assembly recruits COPII proteins to generate the ERES (Gallo et al., 2023). Atg13 is dephosphorylated upon autophagy induction and assembles with other Atg proteins, which form LLPS condensates to organize autophagosome formation sites, known as pre-autophagosome structures (Fujioka et al., 2020). In contrast, the Sec16 C-IDR does not form LLPS condensates (Gallo et al., 2023). Based on these observations, we suggest that the ability of IDRs to assemble through LLPS is unlikely to be essential for tubule localization, although LLPS assembly of Sed4L has not been examined.

Finally, this study highlights the physiological role of the Sed4 luminal IDR in its assembly at the ERES. The Sed4 mutant lacking the luminal region was previously found to complement the *sed4*Δ phenotypes in a manner comparable to wild-type Sed4, suggesting that the luminal region of Sed4 is not essential for its function (Gimeno et al., 1995; Yorimitsu and Sato, 2023). In contrast, we reported that the interaction of Sed4 with Sec16 is critical for Sed4 function (Yorimitsu and Sato, 2023). All Sed4 chimera constructs with the luminal region examined in Fig. 6 retain the intact cytosolic domain, which ensures their ability to interact with Sec16, suggesting that these constructs are functional for ER export.

Among the IDRs examined here, only the Sec16 C-IDR did not function similar to Sed4L. These results suggest that tubule-sensing mechanisms are not sufficient for Sed4 assembly at the ERES. Recently, it was shown that the disordered luminal regions of the single-spanning ER transmembrane protein IRE1α form LLPS condensates and that the LLPS behavior of the luminal regions is important for IRE1α clustering induced in a response to ER stress (Kettel et al., 2024). Based on these findings, we suggest that, unlike tubule sensing, the ability to undergo LLPS could influence the Sed4 assembly at the ERES. In addition to the interaction with Sec16 through the cytosolic domain, as previously reported (Yorimitsu and Sato, 2023), Sed4 uses the luminal IDR to be enriched in tubules by sensing the specific properties of the ER lumen, which then facilitates its oligomerization, possibly through IDR-mediated LLPS. These processes in the cytosol and lumen might synergistically function in Sed4 assembly at the ERES and proper ERES organization.

## MATERIALS AND METHODS

### Strains and plasmids

The yeast strains and plasmids used in this study are listed in Table S1. The strains were grown at 30°C in YPD medium (2% peptone, 1% yeast extract and 2% glucose) or synthetic medium (0.67% yeast nitrogen base without amino acids and 2% glucose supplemented with the appropriate nutrients).

For plasmid construction, PCR-based gene amplification was performed using the Phusion DNA High-Fidelity Polymerase (New England Biolabs). Artificially synthesized PpSec12L and CgSed4L DNA fragments were purchased from Eurofins Genomics. The DNA Ligation Kit Mighty Mix (TAKARA Bio) or NEBuilder HiFi DNA Assembly Master Mix (New England Biolabs) was used to insert the DNA fragment into the appropriate plasmid.

### Fluorescence microscopy

Cells grown in the synthetic medium to mid-log phase were visualized using an Olympus IX71 microscope equipped with a CSU10 spinning disk confocal scanner (Yokogawa Electric Corporation), as previously described (Yorimitsu and Sato, 2012). Images were acquired using an electron-multiplying, charge-coupled device camera (iXon DV897; Andor Technology). Photoshop (Adobe) and ImageJ software (National Institutes of Health) were used for image analysis and figure preparation.

### Quantification and statistical analysis

Profile plots were generated with line-scan analysis, and the intensity values of each protein were quantified along the plotted lines using ImageJ software. The background value was subtracted from the quantified value, and the obtained values were normalized to the maximum value. Microsoft Excel was used to create the intensity profile plots using the obtained values.

Pearson's correlation coefficient was calculated using the ImageJ JACoP plugin. All statistical analyses were performed using Microsoft Excel. Data are plotted as mean±s.d. *P*-values were calculated using an unpaired two-tailed Welch's *t*-test.

## Supplementary Material



10.1242/joces.263696_sup1 Supplementary information
