# Supplementary material for: Potential ER tubular lumen sensing by intrinsically disordered regions
Source: J Cell Sci. 2025 Mar 12;138(5):JCS263696. doi: 10.1242/jcs.263696 (PMC11959615; doi:10.1242/jcs.263696)
Supplement: Supplementary information [file joces-138-263696-s1.pdf]

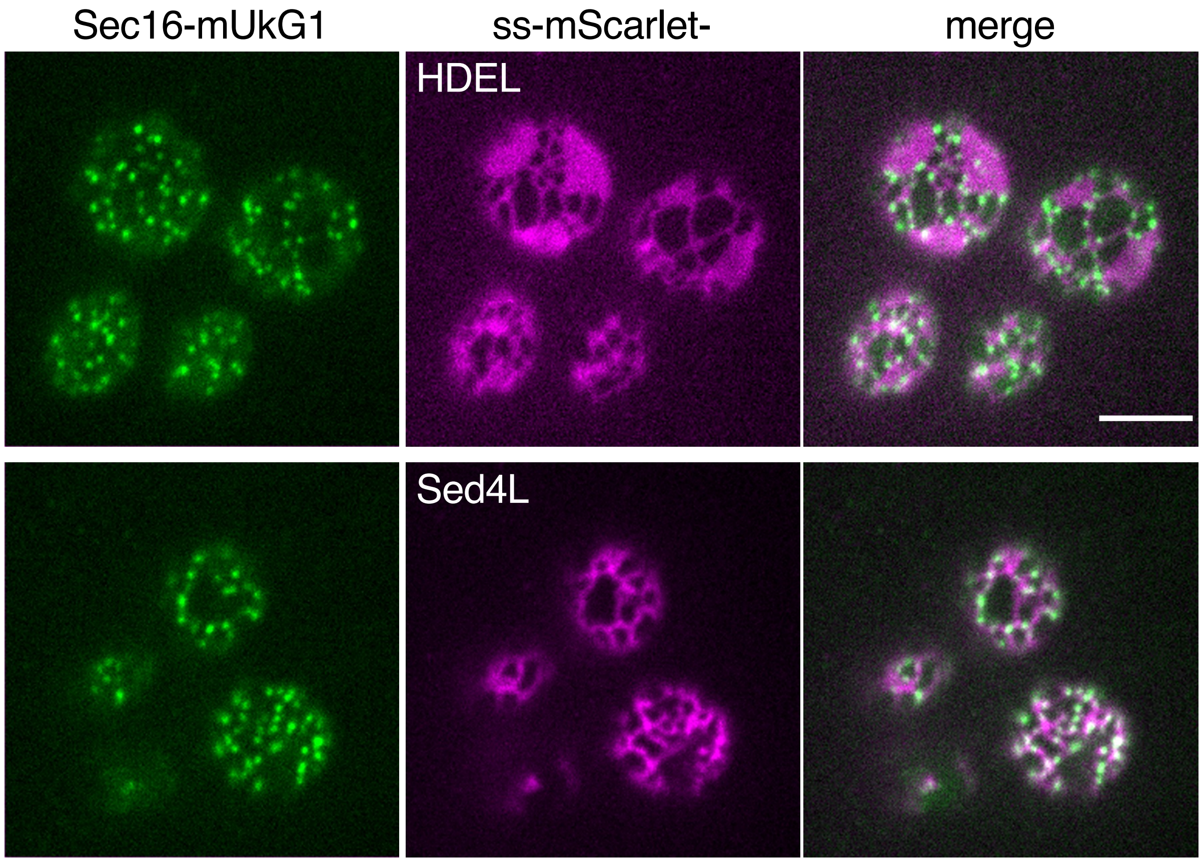

Fig. S1. Translocation of Sed4L into the ER lumen does not affect ERES.
*rtn1*Δ *rtn2*Δ *yop1*Δ cells expressing Sec16-mUkG1 with ss-mScarlet-HDEL, or ss-mScarlet-Sed4L were grown to a mid-log phase. Images were obtained using a confocal fluorescence microscope by focusing on the periphery of the cells. White arrows represent ER sheets. The intensity profile plots along the white dashed arrows in the merged images are shown in the right panels Scale bar: 4 µm. Images are representative of three repeats.

Journal of Cell Science • Supplementary information

Fig S2

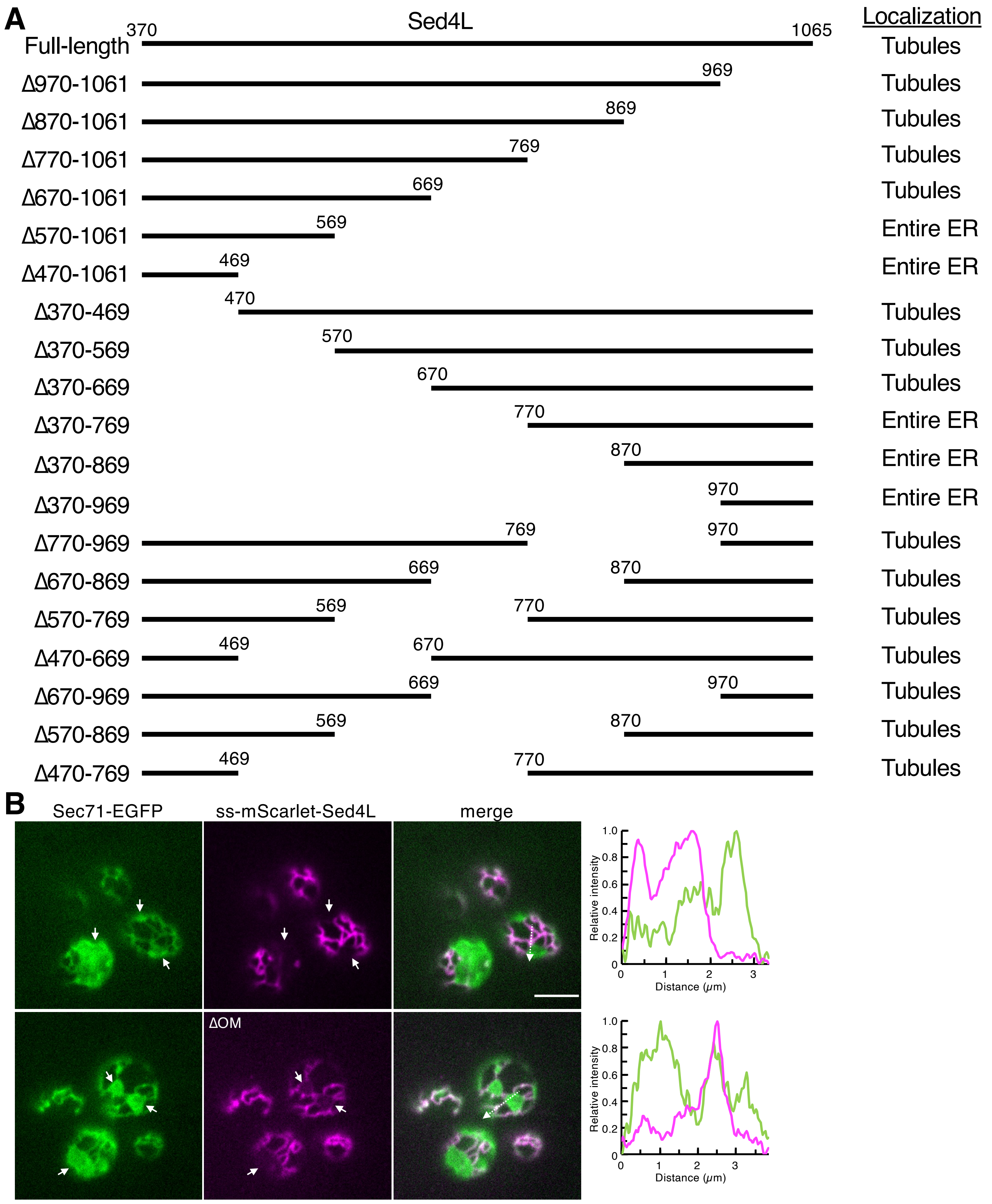

Fig. S2. Whole or multiple parts of Sed4L are required for tubule localization.
*rtn1*Δ *rtn2*Δ *yop1*Δ cells expressing Sec71-EGFP with ss-mScarlet-Sed4L, and its truncation constructs indicated (A) or ss-mScarlet-Sed4LΔOM (B) were grown to a mid-log phase. Images were obtained using a confocal fluorescence microscope by focusing on the periphery of the cells. In (A), schematic diagrams of the truncated region of the luminal domain of Sed4L and, based on the images obtained from three independent experiments, the domain of the ER where each truncation mutant localizes is shown on the right. In (B), white arrows represent the ER sheets and the intensity profile plots along a white dashed arrow in the merged images are shown in the right panels. Scale bar: 4 μm. Images are representative of three repeats.

Journal of Cell Science • Supplementary information

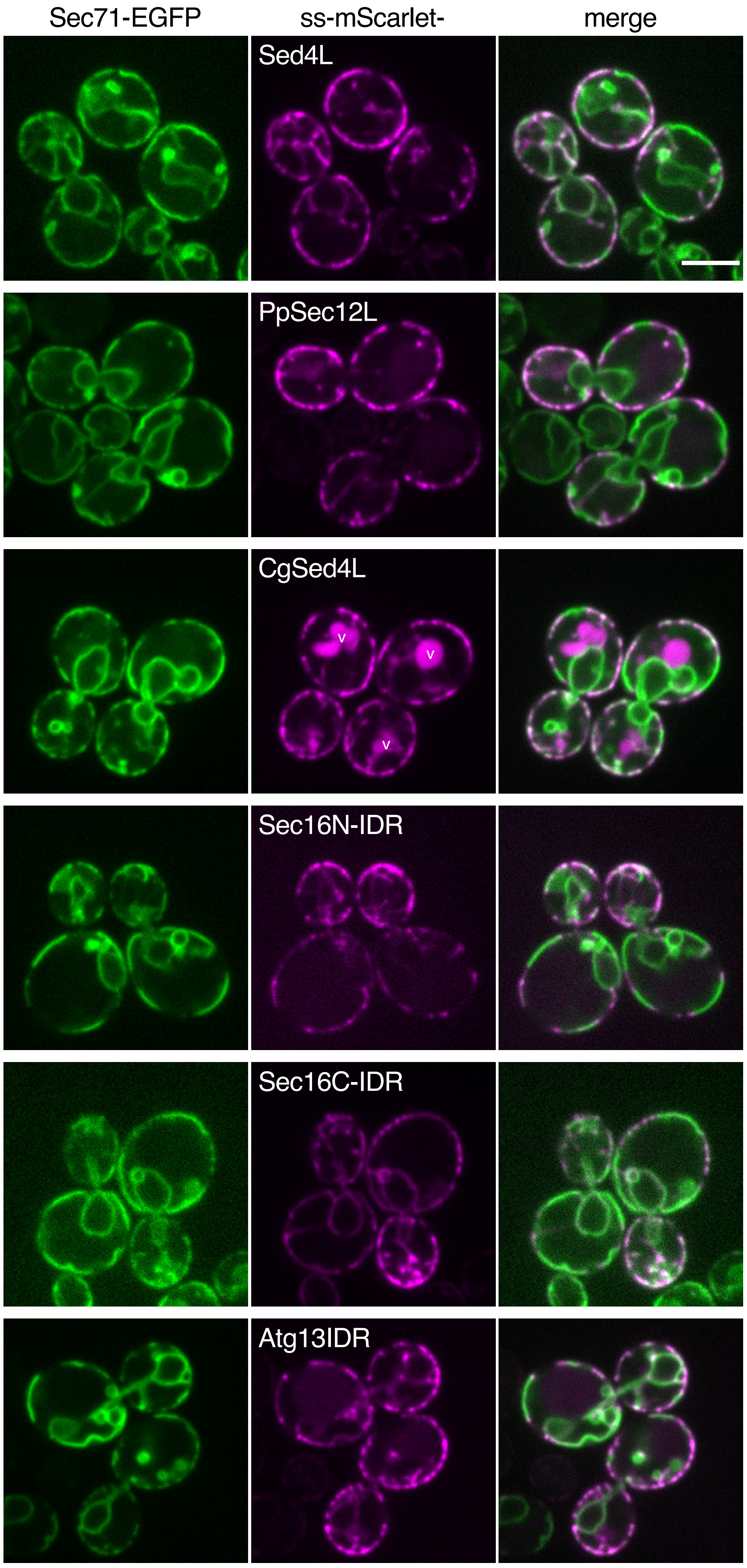

Fig. S3. CgSed4L is partially transported to the vacuole.
Images of cells with ss-mScarlet-Sed4L, ss-mScarlet-PpSec12L, or ss-mScarlet-CgSed4L, and cells with ss-mScarlet-Sec16N-IDR, ss-mScarlet-Sec16C-IDR, or ss-mScarlet-Atg13IDR were the same as those shown in Figs 4C and 5B, respectively, but were observed under a confocal fluorescence microscope by focusing on the center of the cell. Vacuoles are represented by “V”. Scale bar: 4 µm. Images are representative of three repeats.

Journal of Cell Science • Supplementary information

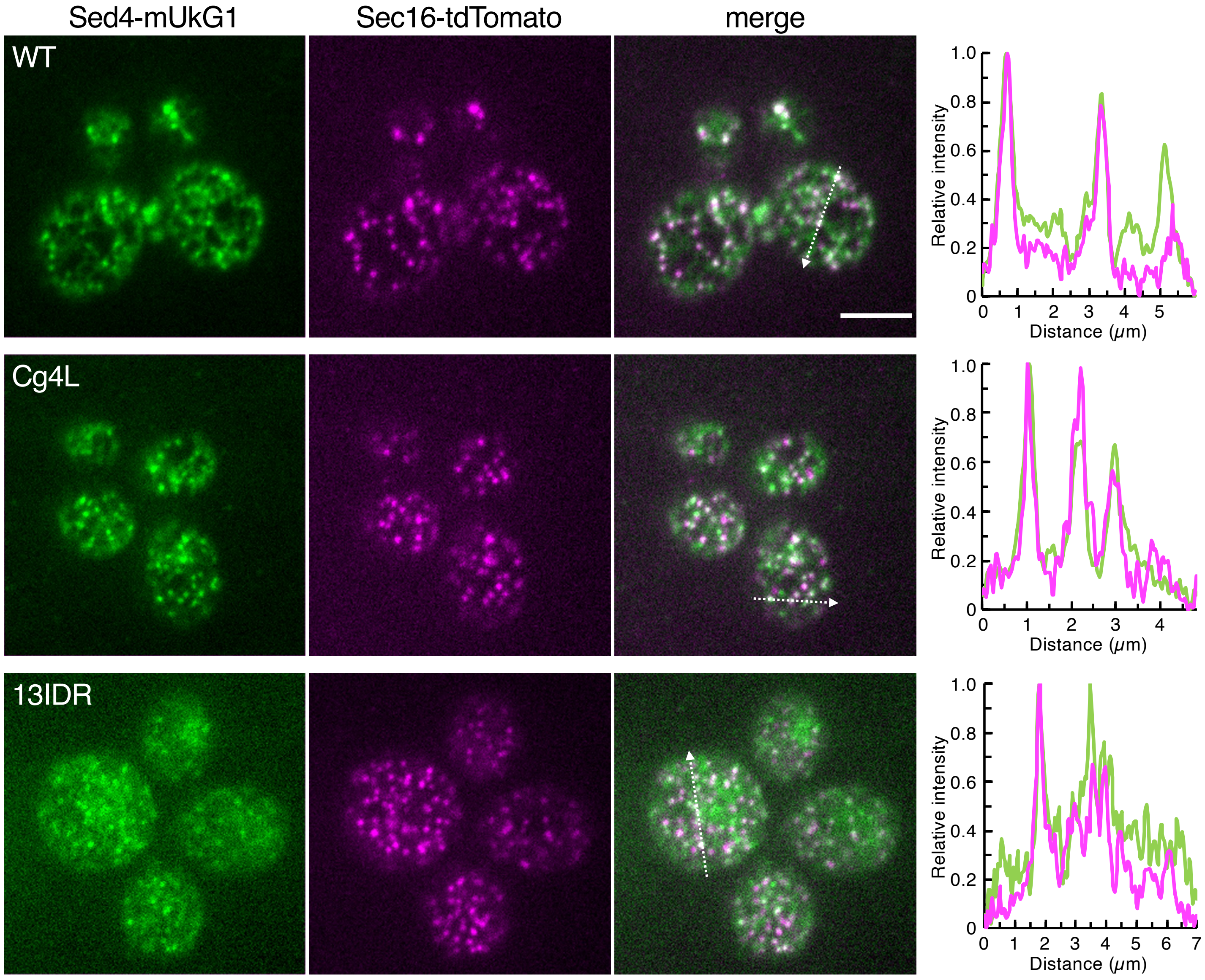

Fig. S4. The CgSed4 luminal region and Atg13IDR act as Sed4L.
Cells expressing Sec16-tdTomato with Sed4-mScarlet, $Sed4^{Cg4L}$-mScarlet, or $Sed4^{13IDR}$-mScarlet were grown to a mid-log phase. Images were obtained using a confocal fluorescence microscope by focusing on the periphery of the cells. The intensity profile plots along the white dashed arrows in the merged images are shown in the right panels. Scale bar: 4 μm. Images are representative of three repeats.

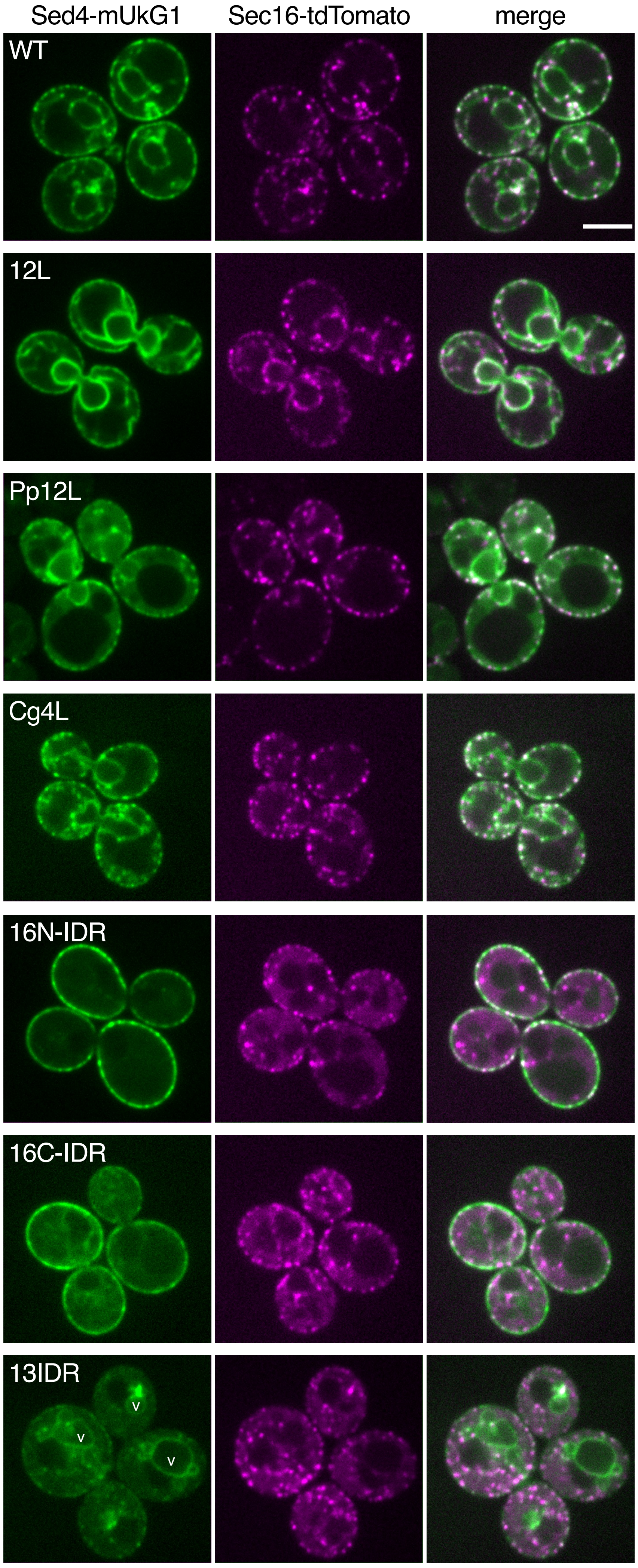

Fig. S5. Sed4^{13IDR} is partially missorted to the vacuolar membrane.
Images of cells with Sed4-mUkG1, Sed4^{12L}-mUkG1, Sed4^{Pp12L}-mUkG1, Sed4$^{16N\text{-}IDR}$-mUkG1, or Sed4$^{16C\text{-}IDR}$-mUkG1, and cells with Sed4^{Cg4L}-mUkG1, or Sed4^{13IDR}-mUkG1 were the same as those shown in Figs 6B and S4, respectively, but were observed under a confocal fluorescence microscope by focusing on the center of the cell. Vacuoles are represented by "V". Scale bar: 4 µm. Images are representative of three repeats.

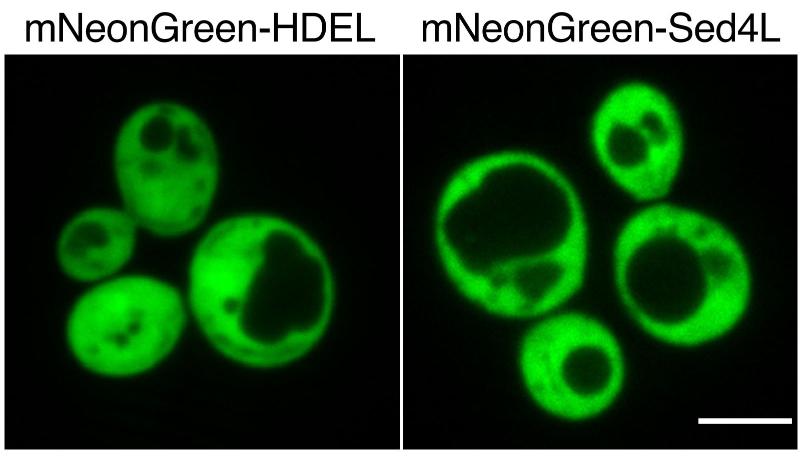

Fig. S6. Sed4L does not localize to the ER but diffuses in the cytosol.
*rtn1*Δ *rtn2*Δ *yop1*Δ cells expressing mNeonGreen-HDEL, or mNeonGreen-Sed4L were grown to a mid-log phase. Images were obtained using a confocal fluorescence Scale bar: 4 μm. Images are representative of three repeats.

Table S1. Yeast strains and plasmids used in this study.

Strain name	Genotype	Figure	Source
YTY207	MATα *ura3-52 lys2-801 ade2-101 trp1-Δ63 his3-Δ200 leu2-Δ1 ADE2 rtn1Δ::NAT rtn2Δ::HIS3 yop1Δ::KAN*	1BC, 2BC, 3AB, 4C, 5B, S2AB, S3, S6	Yorimitsu and Sato (2023)
YTY429	MATα *ura3-52 lys2-801 ade2-101 trp1-Δ63 his3-Δ200 leu2-Δ1 ADE2 sec16Δ::KAN sed4Δ::HIS3* pTYY55	6BC, S4, S5	Yorimitsu and Sato (2023)
YTY432	MATα *ura3-52 lys2-801 ade2-101 trp1-Δ63 his3-Δ200 leu2-Δ1 ADE2 sec16Δ::KAN* pTYY56	S1	This study

Plasmid name	Vector	Description	Figure	Source
pRS314		*TRP1*, *CEN*		Sikorski and Hieter (1989)
pRS316		*URA3*, *CEN*		Sikorski and Hieter (1989)
pSec71-GFP (314)	pRS314	P_{SEC71}_SEC71-EGFP	1BC, 2BC, 3AB, 4C, 5B, S2AB, S3	Yorimitsu and Sato (2023)
pSed4-mScarlet (316)	pRS316	P_{SEC12}_SEC12-mUkG1	1B, 6BC, S4, S5	Yorimitsu and Sato (2023)
pSed4^{12C}-mScarlet (314)	pRS316	P_{SED4}_SEC12$^{1-354}$-SED4$^{347-1065}$-mScarlet-I	1BC	Yorimitsu and Sato (2023)
pSed4ΔL^{12C} -mScarlet (314)	pRS316	P_{SED4}_SEC12$^{1-354}$-SED4$^{347-543}$-mScarlet-I	1BC	Yorimitsu and Sato (2023)
pSec12-mScarlet (316)	pRS316	P_{SEC12}_SEC12-mScarlet-I	1BC, 2BC	Yorimitsu and Sato (2023)
pmScarlet-Sec22 (316)	pRS316	P_{SED4}_mScarlet-I-SEC22	2BC	This study
pSec12-mScarlet-Sed4L (316)	pRS316	P_{SEC12}_SEC12-mScarlet-I-SED4$^{370-1065}$	2BC	This study
pmScarlet-Sec22-Sed4L (316)	pRS316	P_{SED4}_mScarlet-I-SEC22-SED4$^{370-1065}$	2BC	This study
pss-mScarlet-HDEL (316)	pRS316	P_{SED4}_KAR2$^{1-62}$-mScarlet-I-HDEL	3AB, S1	This study
pss-mScarlet-Sed4L (316)	pRS316	P_{SED}_-KAR2$^{1-62}$-mScarlet-I-SED4$^{370-1065}$	3AB, 4C, 5B, S1, S2AB, S3	This study
pss-mScarlet-Sec12L (316)	pRS316	P_{SED4}_KAR2$^{1-62}$-mScarlet-I-SEC12$^{375-471}$-HDEL	3AB	This study
pss-mScarlet-Sed4LΔ970-1061 (316)	pRS316	P_{SED4}_KAR2$^{1-62}$-mScarlet-I-SED4$^{370-969/1062-1065}$	S2A	This study
pss-mScarlet-Sed4LΔ870-1061 (316)	pRS316	P_{SED4}_KAR2$^{1-62}$-mScarlet-I-SED4$^{370-869/1062-1065}$	S2A	This study
pss-mScarlet-Sed4LΔ770-1061 (316)	pRS316	P_{SED4}_KAR2$^{1-62}$-mScarlet-I-SED4$^{370-769/1062-1065}$	S2A	This study
pss-mScarlet-Sed4LΔ670-1061 (316)	pRS316	P_{SED4}_KAR2$^{1-62}$-mScarlet-I-SED4$^{370-669/1062-1065}$	S2A	This study
pss-mScarlet-Sed4LΔ570-1061 (316)	pRS316	P_{SED4}_KAR2$^{1-62}$-mScarlet-I-SED4$^{370-569/1062-1065}$	S2A	This study
pss-mScarlet-Sed4LΔ470-1061 (316)	pRS316	P_{SED4}_KAR2$^{1-62}$-mScarlet-I-SED4$^{370-469/1062-1065}$	S2A	This study
pss-mScarlet-Sed4LΔ370-469 (316)	pRS316	P_{SED4}_KAR2$^{1-62}$-mScarlet-I-SED4$^{470-1065}$	S2A	This study
pss-mScarlet-Sed4LΔ370-569 (316)	pRS316	P_{SED4}_KAR2$^{1-62}$-mScarlet-I-SED4$^{570-1065}$	S2A	This study

pss-mScarlet-Sed4LΔ370-669 (316)	pRS316	P_{SED4}_$KAR2^{1-62}$-mScarlet-I-$SED4^{670-1065}$	S2A	This study
pss-mScarlet-Sed4LΔ370-769 (316)	pRS316	P_{SED4}_$KAR2^{1-62}$-mScarlet-I-$SED4^{770-1065}$	S2A	This study
pss-mScarlet-Sed4LΔ370-869 (316)	pRS316	P_{SED4}_$KAR2^{1-62}$-mScarlet-I-$SED4^{870-1065}$	S2A	This study
pss-mScarlet-Sed4LΔ370-969 (316)	pRS316	P_{SED4}_$KAR2^{1-62}$-mScarlet-I-$SED4^{970-1065}$	S2A	This study
pss-mScarlet-Sed4LΔ770-969 (316)	pRS316	P_{SED4}_$KAR2^{1-62}$-mScarlet-I-$SED4^{370-769/970-1065}$	S2A	This study
pss-mScarlet-Sed4LΔ670-969 (316)	pRS316	P_{SED4}_$KAR2^{1-62}$-mScarlet-I-$SED4^{370-669/870-1065}$	S2A	This study
pss-mScarlet-Sed4LΔ570-969 (316)	pRS316	P_{SED4}_$KAR2^{1-62}$-mScarlet-I-$SED4^{370-569/770-1065}$	S2A	This study
pss-mScarlet-Sed4LΔ470-969 (316)	pRS316	P_{SED4}_$KAR2^{1-62}$-mScarlet-I-$SED4^{370-469/670-1065}$	S2A	This study
pss-mScarlet-Sed4LΔ670-969 (316)	pRS316	P_{SED4}_$KAR2^{1-62}$-mScarlet-I-$SED4^{370-669/970-1065}$	S2A	This study
pss-mScarlet-Sed4LΔ770-969 (316)	pRS316	P_{SED4}_$KAR2^{1-62}$-mScarlet-I-$SED4^{370-569/870-1065}$	S2A	This study
pss-mScarlet-Sed4LΔ770-969 (316)	pRS316	P_{SED4}_$KAR2^{1-62}$-mScarlet-I-$SED4^{370-469/770-1065}$	S2A	This study
pss-mScarlet-PpSec12L (316)	pRS316	P_{SED4}_$KAR2^{1-62}$-mScarlet-I-$PpSEC12^{366-1038}$	3B, 4C, S3	This study
pss-mScarlet-CgSed4L (316)	pRS316	P_{SED4}_$KAR2^{1-62}$-mScarlet-I-$CgSED4^{371-1029}$	3B, 4C, S3	This study
pss-mScarlet-Sec16N-IDR (316)	pRS316	P_{SED4}_$KAR2^{1-62}$-mScarlet-I-$SEC16^{2-991}$	3B, 5B, S3	This study
pss-mScarlet-Sec16C-IDR (316)	pRS316	P_{SED4}_$KAR2^{1-62}$-mScarlet-I-$SEC16^{1421-21951}$	3B, 5B, S3	This study
pss-mScarlet-Atg13IDR (316)	pRS316	P_{SED4}_$KAR2^{1-62}$-mScarlet-I-$ATG13^{281-738}$	3B, 5B, S3	This study
pSed4^{12L}-mUkG1 (316)	pRS316	P_{SED4}_$SED4^{1-370}$-$SEC12^{379-471}$-mUkG1	6BC, S5	Yorimitsu and Sato (2023)
pSed4^{Pp12L}-mUkG1 (316)	pRS316	P_{SED4}_$SED4^{1-370}$-$PpSEC12^{366-1038}$-mUkG1	6BC, S5	This study
pSed4^{Cg4L}-mUkG1 (316)	pRS316	P_{SED4}_$SED4^{1-370}$-$CgSED4^{371-1029}$-mUkG1	6C, S4, S5	This study
pSed4$^{16N\text{-}IDR}$-mUkG1 (316)	pRS316	P_{SED4}_$SED4^{1-370}$-$SEC16^{2-991}$-mUkG1	6BC, S5	This study
pSed4$^{16C\text{-}IDR}$-mUkG1 (316)	pRS316	P_{SED4}_$SED4^{1-370}$-$SEC16^{1421-2195}$-mUkG1	6BC, S5	This study
pSed4^{13IDR}-mUkG1 (316)	pRS316	P_{SED4}_$SED4^{1-370}$-$ATG13^{281-738}$-mUkG1	6C, S4, S5	This study
pTYY55	pRS314	P_{SEC16}_SEC16-tdTomato	6BC, S4, S5	Yorimitsu and Sato (2023)
pTYY56	pRS314	P_{SEC16}_SEC16-mUkG1	S1	Yorimitsu and Sato (2023)
pss-mScarlet-Sed4LΔOM (316)	pRS316	P_{SED4}_$KAR2^{1-62}$-mScarlet-I-$SED4^{\Delta OM}$	S2B	This study
pmNeonGreen-HDEL (316)	pRS316	P_{TDH3}_ mNeonGreen-HDEL	S6	This study
pmNeonGreen-HDEL (316)	pRS316	P_{TDH3}_ mNeonGreen- $SED4^{370-1065}$	S6	This study

Supplementary references

Sikorski, R. S. and Hieter, P. (1989). A system of shuttle vectors and yeast host strains designed for efficient manipulation of DNA in *Saccharomyces cerevisiae*. *Genetics* **122**, 19-27. doi:10.1093/genetics/122.1.19

Yorimitsu, T. and Sato, K. (2023). Sec16 and Sed4 interdependently function as interaction and localization partners at ER exit sites. *Journal of Cell Science* **136**, jcs261094. doi:10.1242/jcs.261094.

Journal of Cell Science • Supplementary information
