# Supplementary material for: Potential ER tubular lumen sensing by intrinsically disordered regions
Source: J Cell Sci. 2025 Mar 12;138(5):JCS263696. doi: 10.1242/jcs.263696 (PMC11959615; doi:10.1242/jcs.263696)
Supplement: Supplementary file 1 [file joces-138-263696_review_history.pdf]

Journal of Cell Science

Potential ER tubular lumen sensing by intrinsically disordered regions

Tomohiro Yorimitsu and Ken Sato
DOI: 10.1242/jcs.263696

Editor: Tamotsu Yoshimori

Review timeline

Original submission: 3 November 2024
Editorial decision: 17 December 2024
First revision received: 27 January 2025
Accepted: 3 February 2025

Original submission

First decision letter

MS ID#: jcs.263696

MS TITLE: ER tubular lumen-sensing intrinsically disordered regions

AUTHORS: Tomohiro Yorimitsu; Ken Sato

ARTICLE TYPE: Research Article

Dear Dr Sato,

We have now reached a decision on the above manuscript.

To see the reviewers' reports and a copy of this decision letter, please go to:

As you will see, although Reviewer 1 is quite positive, both reviewers raise a number of substantial criticisms that prevent me from accepting the paper at this stage. They suggest, however, that a revised version might be acceptable if you can address their concerns. If you believe you can satisfactorily address the criticisms in a revision, I would be pleased to consider a revised manuscript. We would then send it back to the reviewers for further evaluation.

Comments from the Reviewers:

Reviewer 1: In this study, Yorimitsu and Sato examined the role of the luminal domain of yeast Sed4, which is intrinsically disordered in the most part, and showed that this IDR nature functions to sense the negative curvature of tubular membranes from inside. The sensing does not require the transmembrane domain. Even when the Sed4 luminal domain (Sed4L) alone was translocated into the ER lumen, it was localized to tubules. Sed4-like proteins that have similar luminal domains also showed tubule sensing properties. Surprisingly, not only these Sed4-like proteins but also some IDR domains from unrelated proteins such as cytosol-localized Sec16 and Atg13 appeared to show similar properties.

This is a very interesting paper. Yeast Sed4 has been mysterious for a long time. Its cytosolic domain is very similar to that of Sec12, GEF for Sar1 GTPase, but does not have the GEF activity. Sed4 is not essential for growth, but nevertheless it is involved in the assembly of ER exit sites (ERES). Unlike Sec12, which has a relatively short and dispensable luminal domain, Sed4 has a very large luminal domain that is heavily O-glycosylated.

© 2025. Published by The Company of Biologists under the terms of the Creative Commons Attribution License (https://creativecommons.org/licenses/by/4.0/).

The data shown in the paper are very impressive. The property of Sed4L (and some other IDRs) to accumulate in the tubular region of the ER looks convincing. This work is probably the first to show sensing of negative membrane curvature in the luminal side of organelles. While it is amazing and impactful by itself, several simple questions arose in my mind. First, what is the molecular mechanism of IDR to sense negative curvature? IDRs shown in the paper do not seem to have conserved amino acid sequences. Beside Sed4-like proteins, IDRs from totally unrelated Sec16 and Atg13 also appeared to sense tubules from inside. Are there any common features among them? How about the IDRs that are known to sense positive curvature from the cytosolic side? More examples will be needed to consider how IDRs can recognize negative curvature. The authors argued that the luminal domains of Sed4-like proteins have an ability to assembly at the ERES but IDRs of Sec16 and Atg13 do not. What causes this difference?

As this paper contains amazing findings and raise a new concept of membrane curvature sensing, I would support publication of the work, but the authors should try more efforts to answer the above questions.

Others are minor comments.

Awkward English statements are found at several places. Careful proofreading by a native speaker or AI is strongly recommended.

Fig. 2 in the middle row. Sec4L should read Sed4L.

Reviewer 2: SUGGESTIONS TO AUTHORS
In this report, the authors provided some evidence that intrinsically disordered regions (IDRs) are critical for ER tubule localization. The experiments are conducted reasonably well and the results are convincing to some extent. On the other hand, mechanistic insights into how IDRs target ER tubule is weak.

The authors might find the following specific comments useful to improve the study.
(1) Image analysis is essential performed by line-scan plot, which is not satisfactory. The authors should quantitate the colocalization by the Pearson's correlation coefficients or other means.
(2) In Figure 5, several fusion proteins (12L, Pp12L, and 16N-IDR), were targeted to the ERES. This reviewer is curious if these fusion proteins are functional for the ER exit traffic.
(3) There are a number of fusion proteins used in the study. Their domain structures should be provided as illustration. It was indeed a pain to read this manuscript without illustrations.
(4) line 19: "or" should be better than "and".
(5) line 22: The sentence is misleading.
(6) line 127: "Therefore" appears too strong. The reviewer suggests "In line with".
(7) line 140: The Sed4L sequence is tagged directly to the very C-terminal of Sec12-mScarlet and mScarlet-Sec22? Their luminal domains remain intact? As pointed above, the illustration of the constructs greatly helps this reviewer understand the present work.
(8) Figure 1B: "-Sed4L" is confusing.
(9) line 243: "Therefore" appears strong. The reviewer suggests "In line with".
(10) The study suggests that IDRs senses negative membrane curvature. However, the direct evidence is lacking. The authors should tone down their proposal. The title is not appropriate. It is possible that some lipids or trans-membrane proteins are sorted to ER tubule first, to which proteins with IDRs have the affinity.

First revision

Author response to reviewers' comments

We thank the reviewers for their thoughtful and constructive comments. We have carefully considered these criticisms and have endeavored to address them by including new data and by rewriting the text to clarify some issues. Additions to the original text are highlighted in yellow

© 2025. Published by The Company of Biologists under the terms of the Creative Commons Attribution License (https://creativecommons.org/licenses/by/4.0/).

in the new resubmitted manuscript. We hope that these revisions will satisfy the reviewers' concerns.

Reviewer 1: In this study, Yorimitsu and Sato examined the role of the luminal domain of yeast Sed4, which is intrinsically disordered in the most part, and showed that this IDR nature functions to sense the negative curvature of tubular membranes from inside. The sensing does not require the transmembrane domain. Even when the Sed4 luminal domain (Sed4L) alone was translocated into the ER lumen, it was localized to tubules. Sed4-like proteins that have similar luminal domains also showed tubule sensing properties. Surprisingly, not only these Sed4-like proteins but also some IDR domains from unrelated proteins such as cytosol-localized Sec16 and Atg13 appeared to show similar properties.

This is a very interesting paper. Yeast Sed4 has been mysterious for a long time. Its cytosolic domain is very similar to that of Sec12, GEF for Sar1 GTPase, but does not have the GEF activity. Sed4 is not essential for growth, but nevertheless it is involved in the assembly of ER exit sites (ERES). Unlike Sec12, which has a relatively short and dispensable luminal domain, Sed4 has a very large luminal domain that is heavily O-glycosylated.

The data shown in the paper are very impressive. The property of Sed4L (and some other IDRs) to accumulate in the tubular region of the ER looks convincing. This work is probably the first to show sensing of negative membrane curvature in the luminal side of organelles. While it is amazing and impactful by itself, several simple questions arose in my mind. First, what is the molecular mechanism of IDR to sense negative curvature?

Response:
We thank the reviewer for the positive evaluation. We acknowledge that the mechanistic understanding of how the IDRs specifically localize to ER tubules remains incomplete. As described in Discussion section, we have outlined two mechanistic models currently proposed for positive membrane curvature sensing IDRs (Zeno et al. 2018, 2019). However, as noted, these models do not appear to be apply to the IDRs studied in this study.

One mechanism by which the IDRs preferentially sense positive curvature is based on their conformational entropy. IDRs adopt a wide range of conformational diversity with a high flexibility. When the IDRs are bound to the membrane, the membrane surface restricts their conformational diversity, resulting in a reduction of their conformational entropy. The positively curved membrane surface bends away from the IDRs, allowing them to increase the number of available conformations with higher conformational entropy when present on a positively curved membrane.

The second mechanism involves electrostatic repulsion between the IDRs and lipids on the membrane surface. The negatively charged amino acids of the IDRs exert electrostatic repulsion against the anionic lipid head groups. However, the positive curvature allows separation between the IDRs and lipids on the membrane surface, thereby easing their electrostatic repulsive interaction. These two mechanisms have been proposed to work together for the positive curvature-ending IDRs.

For negatively curved membranes, the situation is more complex. They form a surface that bends toward the IDRs, potentially causing higher conformational restriction and increased electrostatic repulsion compared to the positively curved membrane. Interestingly, unlike the positive curvature-sensing IDRs, the IDRs studied here do not require the transmembrane domain or the membrane binding domain, while like the positive curvature-sensing IDRs, they contain a high number of negatively charged residues and a limited number of positively charged residues. However, despite this similarity, the mechanistic model for the positive curvature sensing IDRs may not fully explain the IDR behavior observed here.

Further studies will be required to elucidate the mechanism underlying the negatively curved tubule-localizing IDRs, as noted below. In light of this, we have revised the manuscript to soften our proposal. In the revised manuscript, we have also discussed alternative models involving the lipids and proteins specifically present in the ER tubules. Although there is currently little information on the potential candidate molecules, we cannot completely

© 2025. Published by The Company of Biologists under the terms of the Creative Commons Attribution License (https://creativecommons.org/licenses/by/4.0/).

exclude the possibility that the IDRs indirectly sense negative curvature through association with these molecules.

As evaluated by the reviewer, we believe that our findings are sufficiently novel and valuable not only for researchers studying COPII trafficking, but also for researchers in other fields, including those investigating IDR function. We also hope that this study will inspire further investigations into the specific properties of the IDRs, including their role in membrane curvature sensing.

IDRs shown in the paper do not seem to have conserved amino acid sequences. Beside Sed4-like proteins, IDRs from totally unrelated Sec16 and Atg13 also appeared to sense tubules from inside. Are there any common features among them?

Response:
While we believe that these proteins share general IDR features, such as flexible conformation and an abundance of negatively charged residues, we have not found other common features among them at this time.

However, we have noticed that the luminal region of the Sed4 family (659-696 a.a.), Sec16N-IDR (990 a.a.), Sec16C-IDR (775 a.a.), and Atg13IDR (458 a.a.) all consist of relatively long amino acid sequences. One possible feature related to their tubule-specific localization behavior is the large size of these IDRs. This idea may be supported by our results with the Sed4L truncation variants shown in Figure S2A, which indicate that the truncated forms of Sed4L with less than 300 a.a. do not appear to sense the tubules.

It is important to note that only six IDRs were examined in this study. As noted below by the reviewer, future investigation of a broader range of IDRs may provide further insight into common features that contribute to their tubule localization. We believe that this is also important to definitively address the reviewer's questions below.

How about the IDRs that are known to sense positive curvature from the cytosolic side? More examples will be needed to consider how IDRs can recognize negative curvature.

Response:
We agree with the need for additional examples to better understand how IDRs can sense tubule properties, including negative curvature. Based on the known mechanistic models of conformational entropy and electrostatic interactions for positive curvature sensing, as explained above, we suggest that these models do not fully account for the IDR behavior observed in this study.

As our study focused on a limited number of IDRs, we acknowledge that further investigation with a broader range of IDRs will be essential to uncover the detailed mechanisms underlying this intriguing observation.

The authors argued that the luminal domains of Sed4-like proteins have an ability to assembly at the ERES but IDRs of Sec16 and Atg13 do not. What causes this difference?

Response:
As mentioned above, we do not yet understand the specific characteristics of the IDRs studied here and cannot explain the difference in their ability to assemble Sed4 at the ERES. However, we have proposed a hypothesis related to the difference in the ability to undergo LLPS in the manuscript.

Previous studies have shown that Sec16N-IDR and Atg13IDR, but not Sec16C-IDR, assemble by forming liquid-like condensates (Fujioka et al., 2020, Gallo et al., 2023). This aligns with our findings that Sec16N-IDR and Atg13IDR act as the luminal region of Sed4 to promote Sed4 assembly at the ERES, whereas Sec16C-IDR does not exhibit this ability.

We fully acknowledge that to further test this hypothesis, it will be important to determine whether the luminal regions of the Sed4 family proteins can form liquid-like condensates. In

© 2025. Published by The Company of Biologists under the terms of the Creative Commons Attribution License (https://creativecommons.org/licenses/by/4.0/).

addition, it remains to be investigated whether other IDRs, including those known to undergo LLPS, can functionally substitute for the luminal region of Sed4 to promote Sed4 accumulation at the ERES. We also expect that these investigations may also provide alternative models to explain the tubule-sensing function.

As this paper contains amazing findings and raise a new concept of membrane curvature sensing, I would support publication of the work, but the authors should try more efforts to answer the above questions.

Response:
We are grateful for the reviewer's supportive comments and recognition of the significance of this work. We hope that our responses adequately address the reviewers' question.

Others are minor comments.

Awkward English statements are found at several places. Careful proofreading by a native speaker or AI is strongly recommended.

Response:
We had the original manuscript proofread by a professional English editing service. For the revised manuscript, we have done further careful proofreading using AI-based tools. We hope that these revisions address the reviewer's concern.

Fig. 2 in the middle row. Sec4L should read Sed4L.

Response:

This has been corrected.

Reviewer 2: SUGGESTIONS TO AUTHORS
In this report, the authors provided some evidence that intrinsically disordered regions (IDRs) are critical for ER tubule localization. The experiments are conducted reasonably well and the results are convincing to some extent. On the other hand, mechanistic insights into how IDRs target ER tubule is weak.

Response:
We thank the reviewer for the valuable comments. We have carefully considered the feedback and revised the manuscript accordingly. We believe that the revisions have improved the manuscript.

We acknowledge that the mechanistic insights into how the IDRs specifically localize to the ER tubules remain incomplete, as the current data do not allow us to definitively address the elements that IDRs sense for this process. While previous studies have elucidated the function of the cytosolic domain of Sed4, this study provides novel and significant insights into the property and function of the luminal domain of Sed4, which had not been characterized in detail before. Interestingly, these findings were also observed for the IDRs.

Our discovery of the tubule-localizing soluble form of the IDRs, which had never been reported before, suggests that the IDRs possess a previously unrecognized ability to sense specific features of the tubular lumen. This allowed us to raise the intriguing possibility that the IDRs may sense the concave membrane curvature, although we acknowledge that the alternative possibilities cannot yet be completely ruled out, as pointed out by the reviewer. However, we believe that the data presented here will be valuable not only to researchers studying COPII trafficking, but also to researchers in other fields, including those investigating IDR function. We also hope that this study provides a foundation and inspiration for further investigation of this unique IDR behavior.

The authors might find the following specific comments useful to improve the study.
(1) Image analysis is essential performed by line-scan plot, which is not satisfactory. The authors should quantitate the colocalization by the Pearson's correlation coefficients or other means.

© 2025. Published by The Company of Biologists under the terms of the Creative Commons Attribution License (https://creativecommons.org/licenses/by/4.0/).

Response:
We appreciate the reviewer's suggestion and have calculated Pearson's correlation coefficients for a more quantitative analysis of colocalization. The results are shown in Figures 1C, 2C, 3B, and 6C.

(2) In Figure 5, several fusion proteins (12L, Pp12L, and 16N-IDR), were targeted to the ERES. This reviewer is curious if these fusion proteins are functional for the ER exit traffic.

Response:
The Sed4 mutant lacking the luminal region was previously reported to complement the sed4Δ phenotypes comparable to wild-type Sed4, suggesting that the luminal region of Sed4 is not essential for its function (Gimeno et al., 1995; Yorimitsu and Sato, 2023). In addition, we identified the interaction site on the cytosolic domain with Sec16 and showed that the interaction of Sed4 with Sec16 is critical for Sed4 function (Yorimitsu and Sato, 2023). All Sed4 chimera constructs used in Figure 6 (formerly Figure 5) have the luminal region replaced, but retain the intact cytosolic domain, ensuring their ability to interact with Sec16. These observations suggest that these constructs are functional for ER export.

We acknowledge that this point is intriguing and have now included this information in the revised manuscript.

(3) There are a number of fusion proteins used in the study. Their domain structures should be provided as illustration. It was indeed a pain to read this manuscript without illustrations.

Response:
We agree with the reviewer's concern and have now added the schematic diagrams illustrating the domain structures of the fusion constructs in Figures 1A, 2A and 6A. We believe that these illustrations improve the clarity of the manuscript and make it easier to follow.

(4) line 19: "or" should be better than "and".

Response:
We agree with the reviewer's concern. This has been corrected.

(5) line 22: The sentence is misleading.

Response:
We agree with the reviewer's concern and have now revised this sentence in the manuscript to clarify the misleading statement.

(6) line 127: "Therefore" appears too strong. The reviewer suggests "In line with".

Response:
We agree with the reviewer's concern. This has been corrected.

(7) line 140: The Sed4L sequence is tagged directly to the very C-terminal of Sec12-mScarlet and mScarlet-Sec22? Their luminal domains remain intact? As pointed above, the illustration of the constructs greatly helps this reviewer understand the present work.

Response:
Sec12-mScarlet has the mScarlet fusion at the luminal C-terminal end, while mScarlet-Sec22 has mScarlet fusion at the cytosolic N-terminal end. Both Sec12 and Sec22 used for mScarlet fusion are the full-length protein with no missing amino acids, and their luminal and cytosolic regions remain intact. As noted above, we agree with the reviewer's concern regarding the clarity of the constructs and have now added the schematic diagrams illustrating these constructs in the revised manuscript. We believe that the newly added figures and brief descriptions of the constructs in the figure legends, as suggested by the reviewer, enhance the clarity and understanding of this work.

© 2025. Published by The Company of Biologists under the terms of the Creative Commons Attribution License (https://creativecommons.org/licenses/by/4.0/).

(8) Figure 1B: "-Sed4L" is confusing.

Response:
We agree with the reviewer's concern. To indicate the addition of Sed4L to the constructs, "-Sed4L" has now been changed to "+Sed4L".

(9) line 243: "Therefore" appears strong. The reviewer suggests "In line with".

Response:
We agree with the reviewer's concern. This has been corrected.

(10) The study suggests that IDRs senses negative membrane curvature. However, the direct evidence is lacking. The authors should tone down their proposal.

Response:
We agree with the reviewer's comment and acknowledge that the conclusion regarding membrane curvature sensing overstates the results of this work. To address this concern, we have revised this point in the manuscript and, as noted below, expanded the discussion to include the possible involvement of ER proteins and lipids, in addition to negative membrane curvature, as potential contributing factors.

The title is not appropriate.

Response:
We agree that the statement of the negative curvature sensing in the title is not appropriate as a summary of this work. This work clearly demonstrates that the IDRs can specifically localize to the ER tubules when translocated into the ER lumen, which we believe that provides the first evidence suggesting that IDRs may have the potential to sense some specific features of the tubular lumen environment. To more accurately reflect these findings without overstatement, we have now revised the title. We hope that the revised title addresses the reviewer's concern.

It is possible that some lipids or trans-membrane proteins are sorted to ER tubule first, to which proteins with IDRs have the affinity.

Response:
We agree with the reviewer's comment and acknowledge the need to discuss this possibility. We have now addressed this point with relevant details in the revised manuscript.

To test the hypothesis that lipids specifically present in ER tubules may contribute to the tubule localization of the IDRs, we examined the localization of the signal sequence-deficient Sed4L construct mNeonGreen-Sed4L. Given the symmetric distribution of lipids between the inner and outer leaflets of the ER membrane bilayer (Pabst and Keller, 2024; Sprong et al., 2001; van Meer et al., 2008), we speculated that cytosolically expressed mNeonGreen-Sed4L might stain ER tubules if the IDRs bind to tubule-specific lipids. However, like the control mNeonGreen-HDEL, mNeonGreen-Sed4L did not stain any organelle membrane, including the ER, but instead diffused in the cytosol, as shown in new Figure S6.

While the study on the lipids specifically present in the tubules is still very limited, a quantitative lipidomics analysis by Reinhard et al., (2024) provides some insight. It suggests that despite the specific structural features of these domains, the tubular and sheet domains have nearly identical lipid compositions. Therefore, at this time, we have no evidence to support the possibility that Sed4L senses the tubule-specific lipids for its tubule localization.

We also acknowledge the possibility that tubule-localizing proteins may facilitate IDR localization to the tubules. However, based on our current understanding, no ER tubule-localizing proteins that specifically bind to the IDRs within the luminal side have been reported in yeast or other species. In addition, the proteins that have been reported to interact with Sed4 do not exhibit tubule-specific localization. Taken together, further extensive studies are

© 2025. Published by The Company of Biologists under the terms of the Creative Commons Attribution License (https://creativecommons.org/licenses/by/4.0/).

needed to explore the possible contribution of ER proteins, in addition to lipids, to tubule localization of IDRs.

Second decision letter

MS ID#: jcs.263696R1

MS Title: Potential ER tubular lumen-sensing intrinsically disordered regions

Authors: Tomohiro Yorimitsu; Ken Sato

Article Type: Research Article

Dear Dr Sato,

I am happy to tell you that your manuscript has been accepted for publication in Journal of Cell Science, pending standard publication integrity checks.

© 2025. Published by The Company of Biologists under the terms of the Creative Commons Attribution License (https://creativecommons.org/licenses/by/4.0/).
